# Inhibiting peripheral and central MAO-B ameliorates joint inflammation and cognitive impairment in rheumatoid arthritis

**DOI:** 10.1038/s12276-022-00830-z

**Published:** 2022-08-18

**Authors:** Woojin Won, Hyun-Ji Choi, Ji-Young Yoo, Daeun Kim, Tai Young Kim, YeonHa Ju, Ki Duk Park, Hyunbeom Lee, Sang Youn Jung, C. Justin Lee

**Affiliations:** 1 KU-KIST Graduate School of Converging Science and Technology, Korea University, 145 Anam-ro, Seongbuk-gu, Seoul, 02841 Republic of Korea; 2 Center for Cognition and Sociality, Institute for Basic Science (IBS), Daejeon, 34126 Republic of Korea; 3 Department of Biotechnology, CHA University, Seongnam, 13488 Republic of Korea; 4 Convergence Research Center for Diagnosis, Treatment and Care System of Dementia, Korea Institute of Science and Technology (KIST), Seoul, 02792 Republic of Korea; 5 Division of Bio-Medical Science & Technology, KIST School, Korea University of Science and Technology, Seoul, 02792 Republic of Korea; 6 KHU-KIST Department of Converging Science and Technology, Kyung Hee University, Seoul, 02447 Republic of Korea; 7 Center for Advanced Biomolecular Recognition, Korea Institute of Science and Technology, Seoul, 02792 South Korea; 8 Division of Rheumatology, Department of Internal Medicine, CHA Bundang Medical Center, CHA University, Seongnam, 13496 Republic of Korea

**Keywords:** Rheumatoid arthritis, Astrocyte

## Abstract

Rheumatoid arthritis (RA) is an autoimmune disorder characterized by chronic inflammation and the destruction of joints and systemic organs. RA is commonly accompanied by neuropsychiatric complications, such as cognitive impairment and depression. However, the role of monoamine oxidase (MAO) and its inhibitors in controlling neurotransmitters associated with these complications in RA have not been clearly identified. Here, we report that peripheral and central MAO-B are highly associated with joint inflammation and cognitive impairment in RA, respectively. Ribonucleic acid (RNA) sequencing and protein expression quantification were used to show that MAO-B and related molecules, such as gamma aminobutyric acid (GABA), were elevated in the inflamed synovium of RA patients. In primary cultured fibroblast-like synoviocytes in the RA synovium, MAO-B expression was significantly increased by tumor necrosis factor (TNF)-α-induced autophagy, which produces putrescine, the polyamine substrate for GABA synthesis. We also observed that MAO-B-mediated aberrant astrocytic production of GABA was augmented by interleukin (IL)-1β and inhibited CA1-hippocampal pyramidal neurons, which are responsible for memory storage, in an animal model of RA. Moreover, a newly developed reversible inhibitor of MAO-B ameliorated joint inflammation by inhibiting cyclooxygenase (Cox)-2. Therefore, MAO-B can be an effective therapeutic target for joint inflammation and cognitive impairment in patients with RA.

## Rheumatoid arthritis: Potential therapeutic target identified

Inhibiting an enzyme that is upregulated during joint inflammation may prove a valuable therapy for rheumatoid arthritis (RA). As well as causing considerable pain and discomfort in the joints, RA can also trigger neuropsychiatric problems including depression and memory impairment. The monoamine oxidase (MAO) enzyme family is involved in the control of neurotransmitters, and there is evidence that links MAO-B levels with systemic inflammation. C. Justin Lee at Center for Cognition and Sociality, Institute for Basic Science,, Daejeon, South Korea, and co-workers examined the role of MAO-B in RA using patient tissue samples and mouse models. MAO-B and related molecules were upregulated in patients’ inflamed joint tissues. In mice, elevated MAO-B triggered the inhibition of nerve cell activity related to memory storage. A novel drug that inhibits MAO-B reduced RA-related inflammation and cognitive impairment in mice, suggesting a promising approach to treatment.

## Introduction

Rheumatoid arthritis (RA) is a systemic autoimmune disorder that is mainly characterized by joint inflammation and destruction followed by systemic inflammation^1–4^. Joint inflammation and systemic involvement lead to chronic pain and reduced quality of life and life expectancy in patients with RA^1–3^. The specific organs that can be affected in RA include the skin, eye, lung, kidney, and brain^5,6^. Although the data vary between studies, 13–70% of RA patients have other neuropsychiatric disorders, such as depression, anxiety, panic disorder, and cognitive impairment^6–12^. These neurological symptoms are thought to be caused by neuroinflammation, which originates from peripheral inflammation^13^. However, the precise molecular and cellular mechanisms and therapeutic targets of RA and neuropsychiatric disorders remain unclear.

Monoamine oxidases (MAOs), including MAO-A and MAO-B, are enzymes that catalyze the oxidation of monoamines and are bound to the outer mitochondrial membrane in cells of several organs, such as the brain, liver, kidney, and the immune system^14,15^. Previous reports have suggested that MAO inhibitors can alleviate joint symptoms such as pain and stiffness in patients with RA, and there is growing evidence that MAO-B may be associated with systemic inflammation, especially neuroinflammation^16–18^. Moreover, joint inflammation in RA patients is associated with hydrogen peroxide (H_2_O_2_) production, which might be mediated by MAO-B and mitigated by antioxidant administration, suggesting that MAO-B could be an effective therapeutic target for RA^17,19,20^.

Additional evidence of neuroinflammation includes increased levels of inflammatory cytokines such as tumor necrosis factor (TNF)-α, interleukin (IL)-1β, and IL-6 in the cerebrospinal fluid of patients with RA^21^. Similar to the increase in cytokines, astrogliosis and activated microglia were observed in the brain cortex and hippocampus of animal models of RA^22–25^. We have previously reported that in inflammatory conditions such as Alzheimer’s disease, the hippocampus shows an increased level of MAO-B-dependent gamma aminobutyric acid (GABA) release from reactive astrocytes, which is followed by memory impairment^16^. We also reported that intraventricular infusion of IL-1β in the hypothalamus increases astrocytic GABA release and anxiety-like behavior^26^. However, to date, a causal relationship between astrogliosis and cognitive impairment has not been established in RA patients. Therefore, we hypothesized that neuroinflammation induced by RA augments MAO-B-dependent astrocytic GABA, which can cause cognitive impairment in patients with RA.

In this study, we hypothesized that MAO-B exacerbates joint inflammation and induces aberrant astrocytic GABA release in the hippocampus, leading to cognitive impairment in RA. To identify the pathological role of MAO-B in RA, we performed RNA-sequencing, metabolite quantification, cognition-related behavioral tests, whole-cell patch-clamp recordings, and a newly developed MAO-B inhibitor called KDS2010^27^.

## Materials and methods

### Human tissue and sample preparation

Human synovial tissue was obtained from the knee joints of 5 patients with RA and OA who underwent total knee arthroplasty. Patient serum and synovial fluids were also obtained from 10 patients with RA and OA. All samples were collected after informed consent was obtained, and ethical approval was provided by the CHA Bundang Medical Center.

### Primary fibroblast-like synoviocytes

RA fibroblast-like synoviocytes (FLSs) were isolated from the synovial tissue of RA patients. Synovial tissues were washed with sterile PBS and minced in DMEM containing 10% FBS, penicillin (100 U/ml), and streptomycin (100 µg/ml) (Gibco). Trimmed synovial tissues were digested in culture media containing collagenase I and deoxyribonuclease I for 2 h at 37 °C. Homogenized tissues were resuspended in media. The mixture was centrifuged, and the pellet was resuspended in fresh media and cultured at 37 °C in 5% CO_2_.

### Animals and housing

All DBA/1J mice were group-housed in a temperature- and humidity-controlled environment with a 12 h light/dark cycle and had free access to food and water. All animal care and handling was approved by the Institutional Animal Care and Use Committee of the Institute for Basic Science (IBS-2020-005; Daejeon, Korea) and CHA University (IACUC15008; Seongnam, Korea). For the animal model of RA, ex vivo cytokine incubation, IL-1ra injection, and IL-1β infusion experiments, 6- to 8-week-old female DBA/1J mice were used. It has been demonstrated that the female rodent model of RA shows higher susceptibility than the male rodent model of RA due to aberrant T helper responses and robust IFN-γ and IgG2a responses in females^28,29^. Different groups of animals were used for each experiment. To examine the long-term effects of the MAO-B inhibitor KDS2010 (kindly provided by Dr. Ki Duk Park at KIST), CIA mice were administered 10 or 30 mg/kg/day KDS2010 (in water) ad libitum after the second immunization. To investigate the short-term effects of KDS2010, CIA mice that exhibited a clinical score of approximately 8 at 60 days after the first immunization were selected and administered KDS2010 for 1 week.

### Induction of the RA animal model and evaluation of RA severity

DBA/1J mice were used to create a collagen-induced mouse model, as previously described^30^. The mice were immunized with a subcutaneous injection of chicken type II collagen (Sigma-Aldrich), which was dissolved in 50 mM acetic acid and emulsified in an equal volume of complete Freund’s adjuvant (Sigma-Aldrich). After 3 weeks, a booster injection of an equal volume of chicken type II collagen homogenized with incomplete Freund’s adjuvant (Sigma-Aldrich) was administered. RA severity was evaluated by determining the clinical score and paw thickness, as previously described^30^. The clinical arthritis scores (0–4 scale) were evaluated for each limb, and the maximum score was 16. Paw thickness was also measured with a caliper.

Detailed methodologic information is described in the Supplementary Text.

### Statistical analysis

All statistical analyses were performed using GraphPad Prism v.9.1.0. For comparisons between two groups, an unpaired two-tailed Student’s *t* test was used. Comparisons among three or more groups were performed using one-way or two-way analysis of variance (ANOVA) followed by Tukey’s test for multiple comparisons. *P* values < 0.05 were considered statistically significant. The significance level is represented as asterisks (*p < 0.05, **p < 0.01, ***p < 0.001; ns, not significant). All data are presented as the SEM. Detailed information is reported in Supplementary Table 1.

## Results

### RNA profiling of TNF-α-induced FLSs

**Fig. 1:**
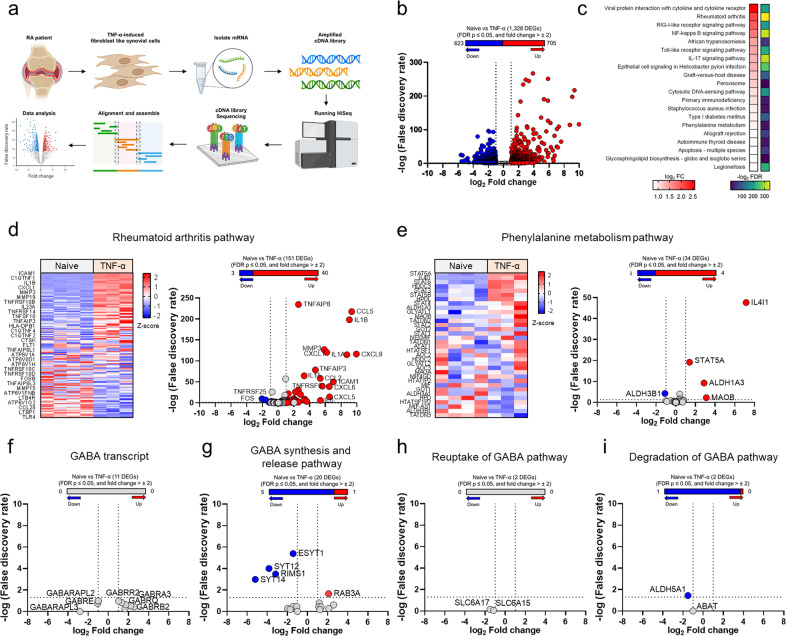
RNA profiling of TNF-α-stimulated fibroblast-like synoviocytes (FLSs). **a** Schematic showing the experimental design for bulk RNA-seq. **b** RNA-seq detected 1328 DEGs between naive (*n* = 4, biologically independent) and TNF-α-induced FLSs (*n* = 3, biologically independent). **c** Top 20 pathways altered in FLSs, which were identified by Partek flow pathway analysis. The log_2_ fold change and –log_2_ FDR are shown. **d** Heatmap (left) and volcano plot (right) of the genes listed in the rheumatoid arthritis pathway. **e** Heatmap (left) and volcano plot (right) of the genes listed in the phenylalanine metabolism pathway. **f**–**i** Volcano plot of the genes listed in GABA-related transcripts. The red circle in the graph indicates significant upregulation; the blue circle indicates significant downregulation; FDR ≤ 0.05, and fold change >± 2.

Inhibition of MAO-B has been suggested to reduce joint symptoms in patients with RA^31,32^. However, the relationship between MAO-B and joint inflammation associated with RA has not yet been established. To determine whether RA pathology correlates with MAO-B, we performed unbiased RNA profiling by RNA-seq of vehicle-treated fibroblast-like synoviocytes (FLSs) derived from RA patients and TNF-α-induced FLSs derived from RA patients (Fig. 1a). TNF-α induces a series of inflammatory signals in FLSs^3,33^. Using Partek flow computational analysis, 1328 differentially expressed genes (DEGs) were detected (Fig. 1b, false discovery rate [FDR] ≤ 0.05, and fold change > ±2). The reads were analyzed using KEGG pathway analysis, which revealed enrichment in rheumatoid arthritis and several inflammatory pathways in TNF-α-stimulated FLSs (Fig. 1c, d). Among the top 20 pathways, pathway analysis also indicated enrichment in the phenylalanine metabolism pathway, in which *Il4i1, Stat5a, Aldh1a3*, and *Mao-b* were significantly upregulated in stimulated FLSs (Fig. 1e). GABA-related pathways were not significantly changed in TNF-α-induced FLSs, except for the GABA release pathway (Fig. 1f–i). These findings suggest that MAO-B but not MAO-A may be highly correlated with joint inflammation in RA.

### MAO-B and GABA are aberrantly expressed in fibroblast-like synoviocytes from RA patients

**Fig. 2:**
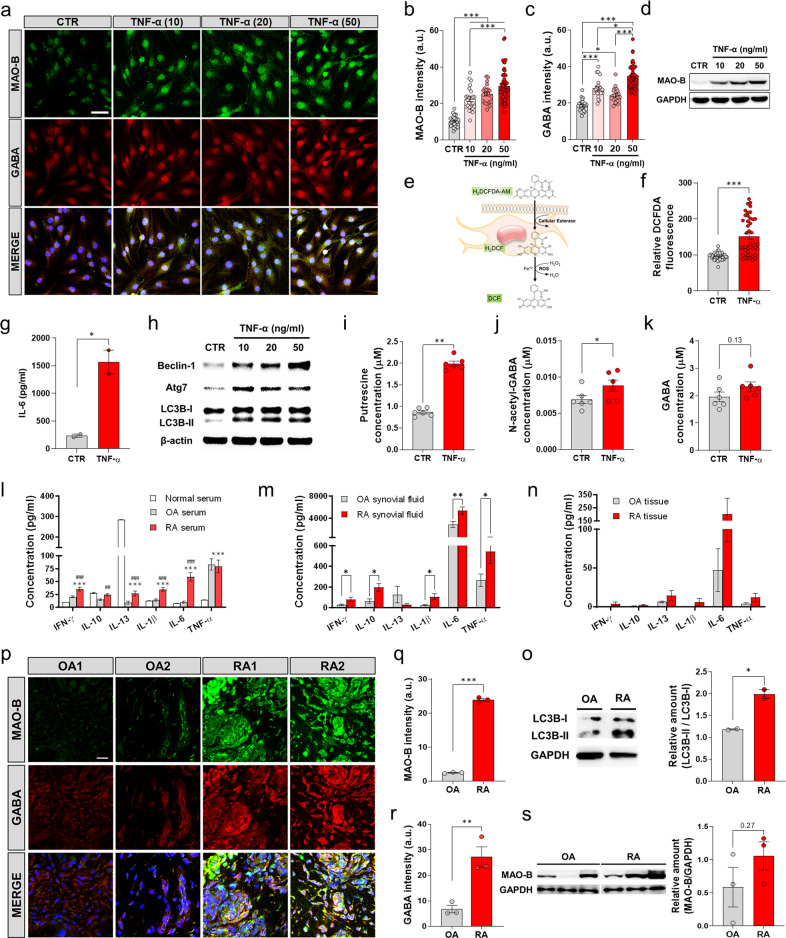
MAO-B and GABA are aberrantly expressed in RA. **a** Representative confocal images of concentration-dependent changes in MAO-B and GABA in response to TNF-α in FLSs (*n* = 3 for each group; scale bar, 50 µm). **b**, **c** Quantification of MAO-B and GABA intensity in FLSs (one-way ANOVA, Tukey’s multiple comparisons test). **d** Western blot showing MAO-B in FLSs. **e**, **f** Schematic image of the DCFDA assay and quantification of H_2_O_2_ in naive and TNF-α-stimulated FLSs (two-tailed unpaired *t* test, *n* = 43 for each group). **g** Quantification of IL-6 expression in naive and TNF-α-stimulated FLSs. **h** Western blot showing activated autophagy markers in TNF-α-activated FLSs. **i**–**k** Metabolite quantification in control and TNF-α-stimulated FLSs (one-way ANOVA, Tukey’s multiple comparisons test). **l** Quantification of IFN-γ, IL-10, IL-13, IL-1β, IL-6, and TNF-α in the serum of normal, OA, and RA patients (one-way ANOVA with Tukey’s multiple comparisons test, *n* = 20, 16, and 16). **m** Quantification of IFN-γ, IL-10, IL-13, IL-1β, IL-6, and TNF-α in the synovial fluid of OA and RA patients (two-tailed unpaired *t* test, *n* = 16 for each group). **n** Quantification of IFN-γ, IL-10, IL-13, IL-1β, IL-6, and TNF-α in the joint tissue of OA and RA patients (two-tailed unpaired *t* test, *n* = 16 for each group). **o** Western blot showing activated autophagy markers in the joint samples of OA and RA patients **p** Representative confocal images showing synovial MAO-B and GABA in OA and RA samples (*N* = 3 for each group; scale bar, 20 µm). **q**, **r** Quantification of synovial MAO-B and GABA (two-tailed unpaired *t* test). **s** Representative Western blot showing MAO-B and GAPDH and the quantification of synovial MAO-B expression (two-tailed unpaired *t* test). Error bars in the graphs indicate the SEM. *P < 0.05; **P < 0.01; ***P < 0.001. Statistical details are provided in Supplementary Table 1.

To determine whether the protein expression of MAO-B and its product GABA corresponds to transcriptional changes in FLSs, we investigated whether MAO-B is correlated with the amount of TNF-α in the RA synovium. We stimulated FLSs with TNF-α (10, 20, or 50 ng/ml) for 24 h, as previously described^34^. Immunocytochemistry showed that TNF-α significantly increased MAO-B and GABA expression in a concentration-dependent manner compared to the control (Fig. 2a–c). In addition, Western blotting demonstrated a similar trend in MAO-B in FLS (Fig. 2d). These results suggest that MAO-B and GABA are correlated with the level of TNF-α. Furthermore, as independent evidence of MAO-B activity, we measured the amount of H_2_O_2_ and found that it was significantly increased by TNF-α (Fig. 2e, f). In addition, IL-6, which is one of the key proinflammatory cytokines associated with RA, was significantly increased by TNF-α (Fig. 2g). Taken together, these results indicate that the expression of MAO-B and the production of GABA and H_2_O_2_ can be induced by TNF-α, which is positively correlated with inflammation^2^.

We next examined whether TNF-α-stimulated FLSs could produce putrescine and N-acetyl-GABA, which are the precursor substrate and direct substrate of MAO-B, respectively, and enhance the forward reaction of MAO-B, which exacerbates inflammation. Western blotting showed an increase in autophagy activation markers, such as Beclin-1, Atg7, and the LC3B-II/LC3B-I ratio (Fig. 2h), suggesting that autophagy activation positively correlates with TNF-α. To examine whether TNF-α increases the substrates, we used electrospray ionization liquid chromatography with mass spectrometry (ESI–LC–MS/MS)-based metabolite quantification. We found that TNF-α significantly increased putrescine, N-acetyl-GABA, and GABA levels (not significant) (Fig. 2i–k), suggesting that GABA synthesis through the putrescine degradation pathway might correlate with TNF-α-induced inflammation. Taken together, these results suggest a positive correlation between autophagy and MAO-B and that MAO-B products, either GABA or H_2_O_2_, might exacerbate joint inflammation by upregulating the expression of proinflammatory factors.

### MAO-B and GABA are aberrantly expressed in the synovium of RA patients

To investigate whether the expression of MAO-B and GABA was changed in the joints of human patients, we compared joint tissue samples from patients with RA and osteoarthritis (OA), which is known to be a less inflammatory form of arthritis^35^. First, we examined whether the levels of proinflammatory cytokines were higher in patients with RA than those with OA. To measure the cytokines in serum, synovial fluid, and tissue, we used a magnetic bead-based immunoassay (Luminex). We found a significant increase in proinflammatory cytokines in the serum of RA patients compared to that of OA patients (Fig. 2l), whereas OA patients had normal serum cytokine levels (Fig. 2l), suggesting that OA patients had a low level of inflammation. Moreover, the synovial fluid of RA patients showed a significant increase in proinflammatory cytokines compared to that of OA patients (Fig. 2m). A similar trend was observed in the tissue samples (Fig. 2n). Taken together, these results confirm that RA patients have higher levels of proinflammatory cytokines than OA patients.

We next examined whether different degrees of inflammation between RA and OA correlated with aberrant autophagy. To test this idea, we performed analyzed RA and OA joint tissue samples by Western blot analysis with antibodies against LC3B-II/LC3B-1. We found that compared to the OA synovium, the RA synovium had significantly higher ratios of LC3B-II/LC3B-1 (Fig. 2o). This result suggests that the increase in autophagy in RA might be due to a higher level of inflammation than in OA^36^. To examine whether increased levels of inflammation and autophagy markers are associated with the increase in MAO-B and GABA, immunohistochemistry (IHC) was performed with antibodies against MAO-B and GABA in the joints (Fig. 2p). Notably, we observed that MAO-B and GABA levels were significantly increased in the RA synovium compared to the OA synovium (Fig. 2q, r). In addition, Western blot analysis showed a similar tendency of increased MAO-B protein in RA samples (Fig. 2s). These results indicate that MAO-B and GABA were present in the joints, and their expression positively correlated with the degree of inflammation. Taken together, these results demonstrate that the aberrant levels of MAO-B and GABA in the joint are highly correlated with the severity of inflammation and proinflammatory cytokines, including IL-1β.

### The RA animal model shows MAO-B-dependent inflammation

**Fig. 3:**
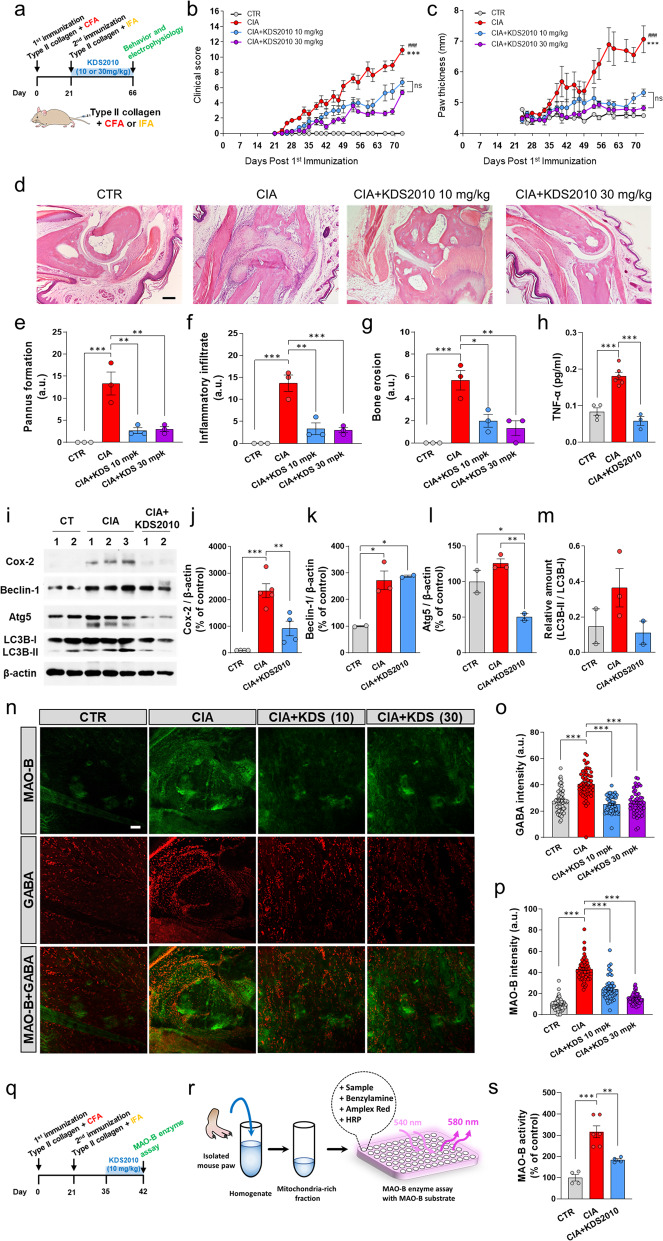
CIA mice show MAO-B-dependent joint inflammation. **a** Schematic timeline of the experiments with CIA mice and KDS2010. **b**, **c** Comparison of arthritis severity clinical scores and paw thickness between control, CIA, and CIA + KD2010 (10 and 30 mg/kg/day) mice (*N* = 2, 7, 5, and 4; two-way ANOVA, Tukey’s multiple comparisons test). A higher score indicates more severe arthritis, and 16 was the highest score. ‘*’ indicates CIA vs. CIA + KDS2010 (10 mg/kg/day) and ‘#’ indicates CIA vs. CIA + KDS2010 (30 mg/kg/day). **d** Representative H&E-stained sections of control, CIA, and CIA + KDS2010 (10 and 30 mg/kg/day) samples (*N* = 3 for each group; scale bar, 200 µm). **e**–**g** Quantification of pannus formation, inflammatory infiltration, and bone erosion based on histological analysis (one-way ANOVA, Tukey’s multiple comparisons test, *N* = 3 for each group). **h** Quantification of TNF-α in the lysates of paw tissues. **i**–**m** Western blot showing activated markers in the lysates of paw tissues from control, CIA, and CIA + KDS2010 mice. **n** Representative confocal images showing synovial MAO-B and GABA in control, CIA, CIA + KDS2010 (10 and 30 mg/kg/day) mice (*N* = 2 for each group; Sale bar, 20 µm). **o**, **p** Quantification of MAO-B and GABA in the synovium of the RA mouse model. **q**, **r** Timeline and schematic image of the MAO-B enzyme analysis of the lysate of paw tissues. **s** MAO-B enzyme analysis of the lysates of paw tissues from control, CIA, CIA + KDS2010 mice (*N* = 2, 3, and 2; one-way ANOVA, Tukey’s multiple comparison test). Error bars in the graphs indicate the SEM. *P < 0.05; **P < 0.01; ***P < 0.001; ns nonsignificant. Statistical details are provided in Supplementary Table 1.

To investigate the role of MAO-B in an animal model of RA, we used KDS2010, a newly developed reversible inhibitor of MAO-B^27,37^. Prolonged treatment with conventional irreversible MAO-B inhibitors, such as selegiline, shows signs of compensatory mechanisms^27^, but KDS2010 circumvents these issues. Furthermore, we used the most frequently used animal model of RA, the collagen-induced arthritis (CIA) mouse model^30^. After the second immunization, CIA mice were orally administered 10 or 30 mg/kg/day KDS2010 ad libitum (CIA + KDS2010) (Fig. 3a). The therapeutic effect of KDS2010 on RA, arthritis clinical score, and paw thickness were measured^30^. The indices of CIA mice started to increase steadily, while 10 and 30 mg/kg/day KDS2010 significantly alleviated these indices (Fig. 3b, c and Supplementary Fig. 1). These results suggest that MAO-B may be involved in the progression of RA.

Next, to confirm the anti-inflammatory effect of KDS2010, we assessed the severity of the disease and joint inflammation. Histological analysis was performed to examine the effect of KDS2010 on joints (Fig. 3d). We found that compared to control mice, CIA mice showed abnormal pannus formation, inflammatory infiltration, and bone erosion (Fig. 3d–g). In contrast, CIA mice that were treated with 10 or 30 mg/kg/day KDS2010 showed significantly reduced indices (Fig. 3d–g), suggesting that MAO-B might be involved in the progression of RA. Moreover, we quantified the levels of the proinflammatory cytokine TNF-α in the lysates of paw tissues from control, CIA, and CIA + KDS2010 mice. TNF-α was significantly increased in CIA mice compared to control mice, and this factor was reduced in CIA mice by the administration of KDS2010 (Fig. 3h). This result indicates that KDS2010 alleviates inflammation in CIA mice.

Next, we investigated whether autophagy-related proteins were increased in relation to inflammation in control, CIA, and CIA + KDS2010 mice. Western blotting showed a significant increase in Cox-2, which is downstream of NF-κB^38,39^, and an increasing trend of autophagy activation, as indicated by Beclin-1, Atg5, and the ratio of LC3B-II/LC3B-1 (Fig. 3i–m). These results suggest that autophagy is associated with the degree of inflammation.

In addition, CIA mice showed significantly higher expression of MAO-B and GABA in joints, which was significantly inhibited by KDS2010 treatment (Fig. 3n–p). Next, we examined whether MAO-B activity corresponded to increased expression of MAO-B. To test this idea, we performed a MAO-B enzyme assay with the lysate of paw tissues from control and CIA mice with or without KDS2010 treatment (Fig. 3q, r). We observed that MAO-B activity was significantly increased in CIA mice compared to control mice, and this effect was reversed by KDS2010 treatment (Fig. 3s). Taken together, these results highlight the importance and therapeutic effect of MAO-B in RA-induced inflammatory arthritis.

### The RA animal model shows MAO-B-dependent cognitive impairment

**Fig. 4:**
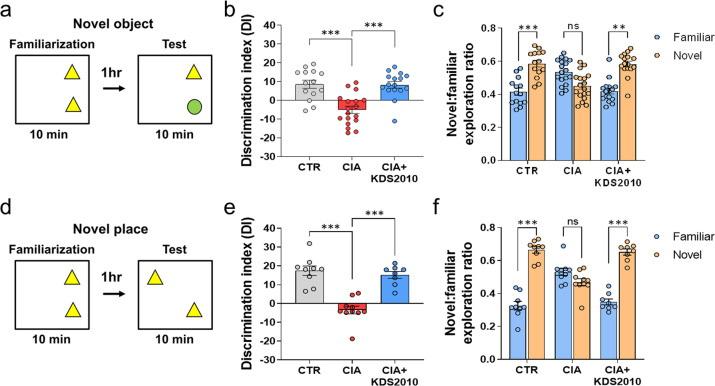
CIA mice show MAO-B-dependent cognitive impairment. **a**, **d** Schematic protocols of the NOR test and NPR test. All objects were positioned in the middle of the first, second, and fourth quadrants of the cage. **b**, **c** The summary results of the NOR test of control, CIA and CIA + KDS2010 (10 mg/kg/day) mice (*N* = 14, 18, and 16; one-way ANOVA, Tukey’s multiple comparisons test). **e**, **f** The summary results of the NPR test of control, CIA and CIA + KDS2010 (10 mg/kg/day) mice (*N* = 9, 10, and 8; one-way ANOVA, Tukey’s multiple comparisons test). Error bars in the graphs indicate the SEM. *P < 0.05; **P < 0.01; ***P < 0.001; ns nonsignificant. Statistical details are provided in Supplementary Table 1.

To determine whether CIA mice develop cognitive impairment similar to that observed in human patients^6–12^, we performed hippocampus-dependent behavioral tests, such as the novel object recognition (NOR) test and novel place recognition (NPR) test (Fig. 4a, d). A higher discrimination index (DI) indicates greater interest in the novel object and better recognition. In the NOR test, CIA mice showed impaired recognition memory, which was ameliorated by KDS2010 (Fig. 4b, c). These results suggest that MAO-B might be responsible for impaired recognition memory in CIA mice.

We performed another hippocampus-dependent recognition task, the NPR test (Fig. 4d). Similar to the results of the NOR test, CIA mice showed impaired spatial recognition memory in the NPR test (Fig. 4e, f), which was reversed by KDS2010 (Fig. 4e, f). To investigate the effect of short-term treatment with KDS2010 during the inflammatory period, we administered KDS2010 for one week to CIA mice (CIA + S. KDS2010), and these mice displayed clinical scores of approximately 8 at 60 days after the first immunization. Similar to long-term treatment with KDS2010, short-term treatment resulted in the recovery of recognition memory (Supplementary Fig. 2a–c). Taken together, these findings suggest that CIA mice show MAO-B-dependent cognitive impairment and that long-term and short-term treatment with KDS2010 can help in the complete recovery of cognitive impairment.

### Aberrant astrocytic GABA inhibition in the hippocampal CA1 region of RA model animals

**Fig. 5:**
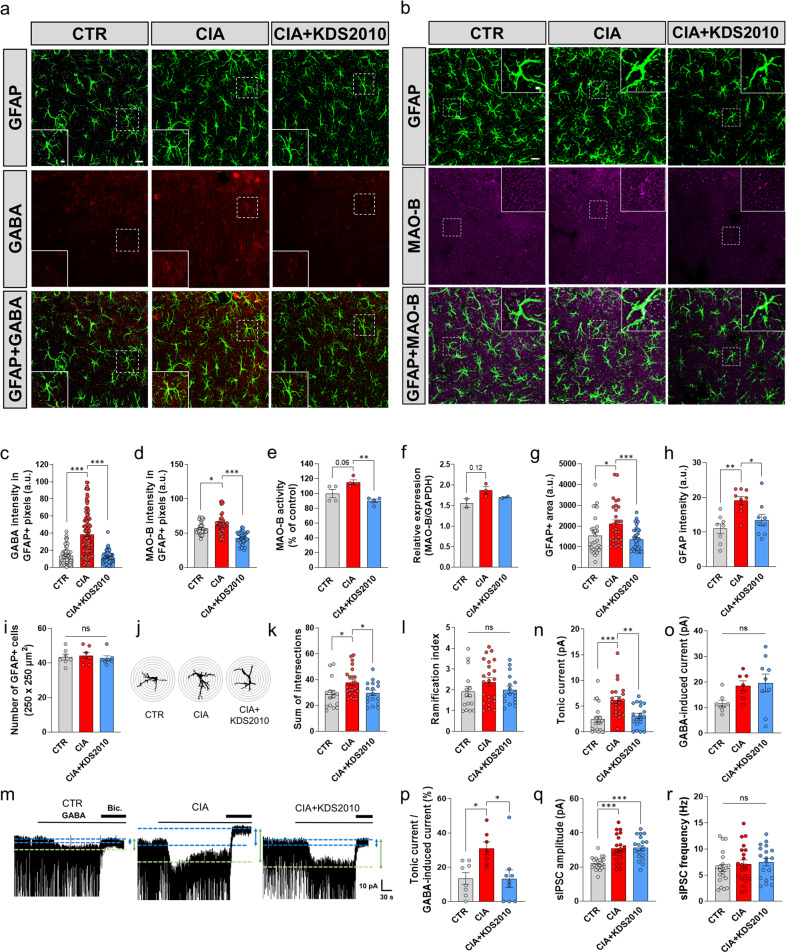
Enhanced inhibitory signaling in the hippocampal CA1 region in CIA mice. **a**, **b** Representative confocal images showing GFAP, GABA, and MAO-B in the CA1-hippocampus (scale bar, 10 µm). **c** GABA intensity in GFAP-positive areas (one-way ANOVA, Tukey’s multiple comparisons test, *N* = 4 for each group; *n* = 53, 104, and 67 for CTR, CIA, and CIA + KDS2010). **d** MAO-B intensity in GFAP-positive pixels (one-way ANOVA, Tukey’s multiple comparisons test, *N* = 3 for each group; *n* = 46, 47, and 41 for CTR, CIA, and CIA + KDS2010). **e** Colorimetric enzymatic analysis of MAO-B activity in hippocampal tissue (one-way ANOVA, Tukey’s multiple comparisons test, *N* = 4 for each group). **f** MAO-B mRNA levels in the hippocampus (one-way ANOVA, Tukey’s multiple comparisons test, *N* = 2 for each group). **g**, **h** Summary graph showing the GFAP-positive area and GFAP intensity (one-way ANOVA, Tukey’s multiple comparisons test, *N* = 4 for each group; *n* = 30, 30, and 36 for **g** and *n* = 8, 9, and 9 for **h**). **i** Number of GFAP-positive cells (*N* = 2 for each group; *n* = 7 for each group). **j** Sholl-analysis image of astrocytes using ImageJ. **k**, **l** The number of intersections and the ramification index of astrocytes (one-way ANOVA, Tukey’s multiple comparisons test, *n* = 15, 23, and 17 for CTR, CIA, and CIA + KDS2010). **m** Representative trace of GABA_A_R-mediated current in the CA1-hippocampus in control, CIA and CIA + KDS2010 mice (10 mg/kg/day). **n** Amplitude of the tonic GABA current (one-way ANOVA, Tukey’s multiple comparisons test, *N* = 5, 6 and 6; *n* = 21, 21, and 19 for CTR, CIA, and CIA + KDS2010). **o** Amplitude of the GABA-induced current (one-way ANOVA, Tukey’s multiple comparisons test, *N* = 3, 3 and 4; *n* = 8, 7, and 9 for CTR, CIA, and CIA + KDS2010). **p** Calculation of the GABA release component (calculated as the tonic current over the GABA-induced full current; one-way ANOVA, Tukey’s multiple comparisons test, *N* = 3, 3, and 4; *n* = 8, 7, and 9 for CTR, CIA, and CIA + KDS2010). **q**, **r** Amplitudes and frequencies of sIPSC amplitudes before bicuculline treatment (one-way ANOVA, Tukey’s multiple comparisons test, *N* = 5, 6, and 6; *n* = 21, 21, and 19 for CTR, CIA, and CIA + KDS2010). Error bars in the graphs indicate the SEM. *P < 0.05; **P < 0.01; ***P < 0.001; ns nonsignificant. Statistical details are provided in Supplementary Table 1.

We found that CIA mice had impairments in object and spatial recognition memory, which is known to be highly associated with the hippocampal CA1 region^40^. We next investigated whether aberrant GABA production in reactive astrocytes in the CA1-hippocampus is responsible for MAO-B-dependent cognitive impairment in CIA mice. To examine astrocytic GABA and MAO-B levels, we performed IHC (Fig. 5a, b). GABA intensity in GFAP-positive cells was significantly increased in CIA mice compared to the control mice (Fig. 5c). In contrast, this increase in GABA was restored to the control level by long-term treatment with KDS2010 in CIA + KDS2010 mice (Fig. 5c), suggesting that aberrant astrocytic GABA was MAO-B-dependent in the CA1-hippocampus. Moreover, MAO-B intensity in GFAP-positive cells was significantly increased in CIA mice compared to control mice, and this effect was reversed by KDS2010 (Fig. 5d). To directly measure the enzyme activity of MAO-B in CIA mice, we performed an enzyme assay. MAO-B activity was increased (but not significantly) in the hippocampal homogenates of CIA mice and was significantly reduced by KDS2010 treatment (Fig. 5e). In addition, a similar increase in MAO-B mRNA was observed (Fig. 5f). These results suggest that MAO-B activity and expression are enhanced in CIA mice.

To test whether these astrocytes show reactive astrogliosis, we performed morphological analyses. We found a significant increase in the GFAP-positive area (Fig. 5g), GFAP intensity (Fig. 5h), and the sum of the intersections based on Sholl analysis, but there were no significant effects on the number of GFAP-positive cells (Fig. 5i) or the ramification index (Fig. 5j–l). However, we did not observe any neuronal death (Supplementary Fig. 3). These results indicate that astrocytes show signs of mild reactive astrogliosis in the CA1-hippocampus of CIA mice. In contrast, KDS2010 in CIA + KDS2010 mice significantly reduced reactive astrogliosis (Fig. 5e–i). Taken together, these results indicate that astrocytes in CIA mice exhibit MAO-B-dependent aberrant GABA and mild reactive astrogliosis.

We next investigated whether MAO-B-dependent astrocytic GABA leads to GABA release and tonic GABA inhibition in the CA1-hippocampus. We measured γ-aminobutyric acid type A receptor (GABA_A_R)-mediated currents in CA1 pyramidal neurons as described previously^41^ (Fig. 5m). We observed that the tonic GABA current in response to treatment with the GABA_A_R antagonist bicuculline (50 µM) was aberrantly increased in CIA mice (Fig. 5n). In contrast, this tonic GABA current was restored to the control level by KDS2010 in CIA + KDS2010 mice (Fig. 5n), indicating that the effect was MAO-B-dependent in CA1-hippocampal pyramidal neurons. To examine whether an alteration in extrasynaptic GABA_A_R or GABA release was responsible for aberrant tonic current, 10 µM GABA was bath-applied to assess the full activation of GABA-induced tonic current (Fig. 5o) as previously described^42–45^. There was no significant difference in the GABA-induced tonic current (Fig. 5o), indicating that the number of extrasynaptic GABA_A_Rs was not significantly different. To further determine whether the release of GABA from astrocytes was changed, we calculated the percentage of tonic GABA current over GABA-induced tonic current. We observed that CIA mice showed significantly increased percentages compared to control mice (Fig. 5p), suggesting that the aberrant tonic current in CIA mice as due to astrocytic GABA release. However, this percentage was reduced by KDS2010, which indicates that inhibiting MAO-B suppresses CA1-hippocampus astrocytic GABA release.

To test whether GABA_A_R-mediated synaptic currents are altered in CIA mice, we measured the amplitude and frequency of GABA_A_R-mediated spontaneous inhibitory postsynaptic currents (sIPSCs). We found a significant increase in amplitude without any effect on the frequency of sIPSCs in CIA mice compared to control mice (Fig. 5q, r), indicating that the number of synaptic GABA_A_Rs was increased, but presynaptic GABA release was unaffected. In contrast to tonic GABA, the amplitude of sIPSCs in CIA mice was not restored by KDS2010 (Fig. 5q), suggesting that the increased number of postsynaptic GABA_A_Rs was independent of MAO-B. Furthermore, the number of GABA-positive cells in the CA1 region in the control, CIA, and CIA + KDS2010 groups was not affected (Supplementary Fig. 4). These results indicate MAO-B-dependent astrocytic GABA release and tonic GABA inhibition. Overall, the IHC and electrophysiology results demonstrated that MAO-B-dependent GABA release from mildly reactive astrocytes but not neuronal GABA in the CA1-hippocampus was responsible for cognitive impairment in CIA mice.

### IL-1β is sufficient for aberrant astrocytic GABA in the CA1-hippocampus

**Fig. 6:**
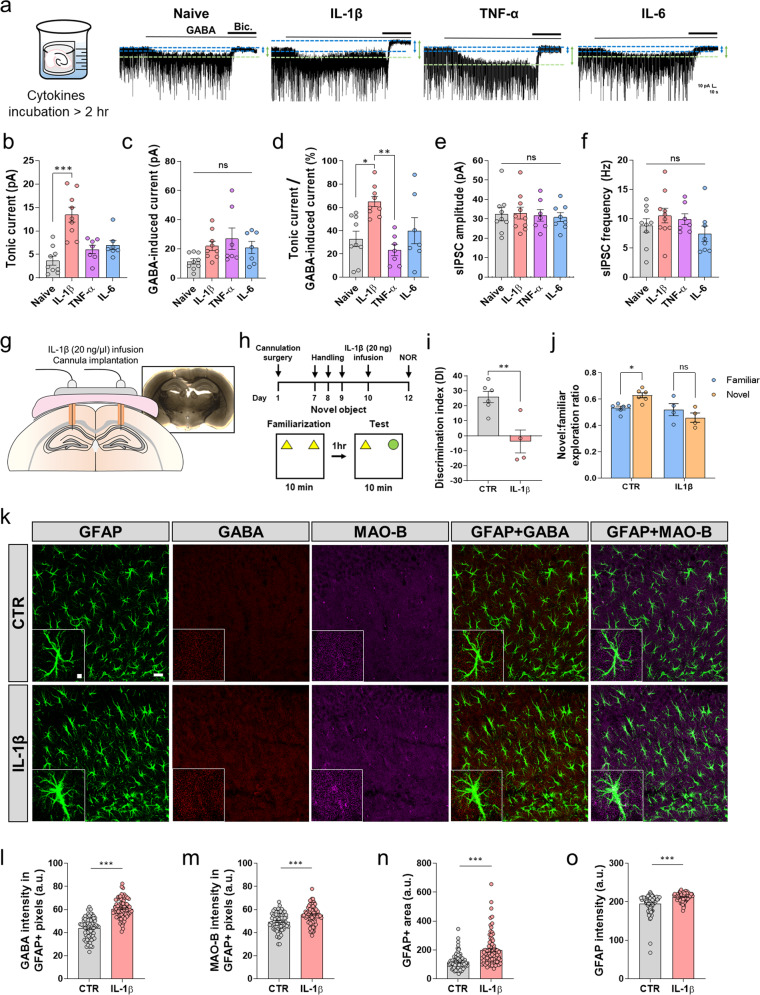
IL-1β is sufficient for aberrant astrocytic GABA in the hippocampus. **a** Schematic image of the incubation and a representative trace of the GABA_A_R-mediated current in the CA1-hippocampus within at least 2 h post of incubation with cytokines, including IL-1β (20 ng/ml), TNF-α (100 ng/ml), and IL-6 (10 ng/ml). **b** Amplitude of tonic current after 2 h of incubation (Kruskal–Wallis test with uncorrected Dunn’s multiple comparisons test, *N* = 3 for each group; *n* = 9, 9, 7, and 7). **c** Amplitude of the GABA-induced current (Kruskal–Wallis test with uncorrected Dunn’s multiple comparisons test, *n* = 9, 9, 7, and 9). **d** GABA release component (calculated as the tonic current over the GABA-induced current, Kruskal–Wallis test with uncorrected Dunn’s multiple comparisons test, *n* = 9, 9, 7, and 7). **e** Amplitude of sIPSCs before bicuculline application (Kruskal–Wallis test with uncorrected Dunn’s multiple comparisons test, *N* = 3 for each group; *n* = 9, 10, 7, and 8). **f** Frequency of sIPSCs before bicuculline application (Kruskal–Wallis test with uncorrected Dunn’s multiple comparisons test, *n* = 9, 10, 7, and 8). **g**, **h** Schematic image of bilaterally implanted guide cannulae and the timeline of IL-1β (20 ng/µl) or vehicle infusion into the hippocampus CA1 and the NOR test. **i**, **j** The summary results of the NOR test in the control and IL-1β infusion groups (*N* = 6 and 4 for control and IL-1β infusion). **k** Representative confocal images showing GFAP, GABA, and MAO-B in the CA1-hippocampus (*N* = 3 for each group; scale bar, 20 µm). **l**, **m** GABA and MAO-B intensity in GFAP-positive areas (*n* = 78 and 82 for control and IL-1β infusion). **n**, **o** The GFAP-positive area and GFAP intensity (*n* = 78 and 82 for each group). Error bars in the graphs indicate the SEM. *P < 0.05; **P < 0.01; ***P < 0.001; ns nonsignificant. Statistical details are provided in Supplementary Table 1.

We next investigated which molecules induced aberrant astrocytic GABA release in the CA1-hippocampus in CIA mice. We hypothesized that this effect may be due to proinflammatory cytokines, such as TNF-α, IL-1β, or IL-6, which are known to be elevated in the hippocampus of CIA mice^22,23^. To test this hypothesis, we incubated brain tissues with cytokines ex vivo (Fig. 6a). We observed that IL-1β significantly increased tonic GABA currents compared to the other treatments (Fig. 6b). There was no significant difference in GABA-induced tonic currents in response to the other cytokines (Fig. 6c). Furthermore, we observed that the percentage of the tonic GABA current over the GABA-induced tonic current significantly increased in response to IL-1β, except for IL-6 (Fig. 6d). These results suggest that, similar to CIA mice, astrocytic GABA release is responsible for aberrant astrocytic tonic current in the IL-1β-incubated CA1-hippocampus. In contrast, the amplitude and frequency of sIPSCs were not significantly different (Fig. 6e, f), indicating that the amplitude of sIPSCs is cytokine-independent. These results suggest that there might be factors other than IL-1β that lead to changes in the amplitude of sIPSCs in CIA mice. Moreover, we found a significant increase in GFAP-positive GABA under IL-1β incubation compared to the naive condition, which was inhibited by KDS2010 administration without affecting GFAP levels (Supplementary Fig. 5a–d). In addition, we found a significant increase in MAO-B activity in a hippocampal culture astrocyte culture in an IL-1β dose-dependent manner, which was reduced by KDS2010 treatment (Supplementary Fig. 5e). Taken together, these results suggest that IL-1β is a key molecule that leads to aberrant astrocytic GABA release and inhibition of the CA1-hippocampus in CIA mice. Next, we investigated whether local IL-1β infusion was sufficient to induce cognitive impairment. We bilaterally implanted guide cannulae and locally infused IL-1β (20 ng/µl) or vehicle into the hippocampal CA1 region in normal mice (Fig. 6g). We found that IL-1β infusion into the CA1 region induced significant cognitive impairment, as measured by the NOR test, similar to that in CIA mice (Fig. 6h–j), indicating that IL-1β is sufficient to induce cognitive impairment. We next investigated whether hippocampal astrocytic GABA and MAO-B were increased by IL-1β infusion. We performed IHC with antibodies against GABA and MAO-B (Fig. 6k–m). We found that astrocytic GABA and MAO-B were significantly increased by IL-1β infusion. Moreover, we observed that the GFAP-positive area and GFAP intensity were significantly increased by IL-1β infusion (Fig. 6n, o).

### IL-1β is necessary for aberrant astrocytic GABA in the CA1-hippocampus

**Fig. 7:**
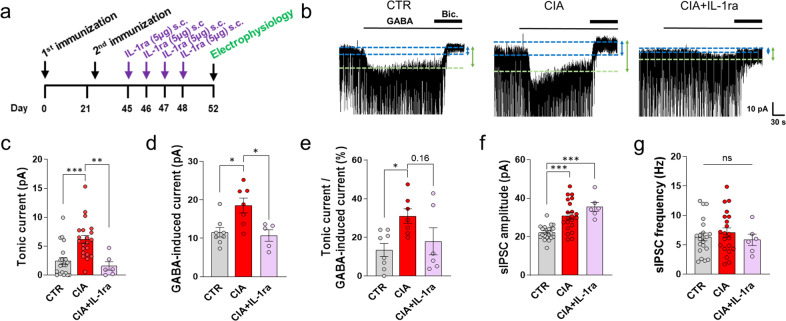
IL-1β is sufficient for aberrant astrocytic GABA in the hippocampus. **a** Timeline of subcutaneous (s.c.) injection of IL-1ra (5 µg) in CIA mice. **b** Representative trace of the GABA_A_R-mediated current in the CA1-hippocampus in control, CIA, CIA + IL-1ra mice. **c** Amplitude of the tonic GABA current (one-way ANOVA, Tukey’s multiple comparisons test, *N* = 5, 6, and 2; *n* = 21, 21, and 5 for CTR, CIA, and CIA + IL-1ra). **d** Amplitude of the GABA-induced current (one-way ANOVA, Tukey’s multiple comparisons test, *N* = 3, 3, and 2; *n* = 8, 7, and 5 for CTR, CIA, and CIA + IL-1ra). **e** Calculation of the GABA release component (calculated as the tonic current over the GABA-induced full current; one-way ANOVA, Tukey’s multiple comparisons test, *N* = 3, 3, and 2; *n* = 8, 7, and 5 for CTR, CIA, and CIA + IL-1ra). **f**, **g** Amplitudes and frequencies of sIPSC amplitudes before bicuculline treatment (one-way ANOVA, Tukey’s multiple comparisons test, *N* = 5, 6, and 2; *n* = 21, 21, and 5 for CTR, CIA, and CIA + IL-1ra). Error bars in the graphs indicate the SEM. *P < 0.05; **P < 0.01; ***P < 0.001; ns nonsignificant. Statistical details are provided in Supplementary Table 1.

We next examined whether IL-1β was necessary for aberrant astrocytic GABA in CIA mice. To test this hypothesis, we subcutaneously injected IL-1 receptor antagonist (IL-1ra), which can easily pass into the brain^46–48^ and neutralize IL-1β, and investigated tonic GABA currents in CIA mice (Fig. 7a). We found that the administration of IL-1ra significantly reduced aberrant tonic GABA currents in CIA mice compared to control mice (Fig. 7b, c). In addition, we observed significant increases in GABA-induced tonic currents in CIA mice compared to control mice (Fig. 7d). On the other hand, the GABA-induced tonic current was significantly decreased in IL-1ra-treated CIA mice, indicating that the number of GABA_A_Rs was decreased by IL-1ra (Fig. 7d). Moreover, the percentage of the tonic GABA current over the GABA-induced tonic current was decreased in IL-1ra-treated CIA mice, suggesting that the reduction in the tonic GABA current as due to a reduction in astrocytic GABA release (Fig. 7e). These results suggest that IL-1β is necessary for aberrant astrocytic GABA. In contrast, the increased amplitudes of sIPSCs in CIA mice was not reversed by IL-1ra, indicating that postsynaptic GABAAR is independent of IL-1β (Fig. 7f). The frequency of sIPSCs was not changed (Fig. 7g). Taken together, these findings indicate that IL-1β is sufficient and necessary to induce aberrant astrocytic GABA in the hippocampal CA1 region.

## Discussion

In this study, we found for the first time that MAO-B was involved in joint inflammation and cognitive impairment in RA. The expression of MAO-B and GABA was identified in the RA synovium, and these substances were produced by TNF-α-stimulated autophagy. Our results suggest that MAO-B can exacerbate arthritis by activating the NF-κB and Cox-2 pathways, and MAO-B-dependent aberrant tonic GABA, which is released from reactive astrocytes in the CA1-hippocampus, leading to cognitive impairment by tonically inhibiting neurons. IL-1β may be the key cytokine that binds and activates interleukin 1 receptor type I (IL-1r1) on astrocytes to cause MAO-B-dependent astrocytic GABA synthesis and release. In particular, our study shows that KDS2010, a recently developed reversible MAO-B inhibitor, significantly ameliorates joint inflammation and cognitive impairment by ameliorating the increase in astrocytic GABA, suggesting that MAO-B is an important therapeutic target in RA (Supplementary Fig. 6).

One of the most important findings in our study is that aberrant astrocytic tonic GABA release in the CA1-hippocampus impairs cognitive function in animal models of RA. Recent studies have shown neuropsychiatric comorbidities associated with neuroinflammation in RA patients, and some studies have reported enhanced IL-1β in the cerebrospinal fluid and increased expression of IL-1β in the hippocampus of RA patients^13,22,23^. Several mechanisms can convey peripheral inflammatory signals to the central nervous system to induce neuroinflammation in the brains of RA patients: (1) circulatory cytokines can enter the brain by volume diffusion; (2) systemic cytokines can infiltrate through the disrupted blood-brain barrier (BBB)^49,50^; (3) direct stimulation of the vagus nerves; and (4) entry through the lymphatic vessels^51^. Numerous reports have shown that peripheral inflammation increases the production of proinflammatory cytokines in the brain, including IL-1β and TNF-α^22,52,53^. In one report, elevated plasma levels of IL-1β contributed to inflammation-induced memory deficits in patients with sepsis-associated encephalopathy^54^. Another study reported that elevated IL-1β levels in the hippocampus were highly linked with memory deficits after orthopedic surgery^55^. Consistent with these reports, we recently reported that IL-1β activates astrocytes to induce reactive astrogliosis and the release of astrocytic GABA through the Best1 channel in the paraventricular nucleus in a depression model^26^. Consistently, our study further shows that MAO-B-dependent astrocytic GABA release was increased in CIA mice and that ex vivo incubation of hippocampal slices with IL-1β but not TNF-α or IL-6 mimicked the brains of CIA mice in a MAO-B-dependent manner. Therefore, this evidence from our current study and other suggests that elevated systemic IL-1β infiltrates the brain and causes aberrant astrocytic GABA production, leading to cognitive impairment in RA.

It has been previously shown that IL-1β induces memory deficits by increasing tonic conductance generated by α5-subunit-containing GABA_A_R (α5-GABA_A_R), which is a major extrasynaptic GABA_A_R expressed in the hippocampus^56^. Consistent with this finding, we showed that IL-1β increased tonic GABA currents, but IL-1β mostly affected astrocytic GABA release without significantly increasing extrasynaptic GABA_A_R. This discrepancy in the mechanism of action might be associated with the fact that tonic GABA currents were recorded after the slices were preincubated with GABA based on a previous study, and alterations in extrasynaptic GABA_A_R but not tonic GABA release could be investigated. In contrast, we used saturating concentrations of GABA after recording the baseline tonic GABA current to monitor tonic GABA release and extrasynaptic GABA_A_R. It is possible that IL-1β enhanced α5-GABA_A_R because we observed increased GABA-induced currents in CIA mice and IL-1β-treated slices (Figs. 5o, 6c). Notably, although KDS2010 was sufficient to ameliorate cognitive impairment, KDS2010 treatment had no effect on GABA-induced currents in CIA mice, indicating that the slight increase in extrasynaptic GABA_A_R was MAO-B-independent. These results strengthen the conclusion that inflammation-induced tonic GABA release from reactive astrocytes is highly associated with memory impairment. However, the remaining roles of α5-GABA_A_R require further investigation.

In our previous studies, we defined reactive astrocytes as mild or severe reactive astrocytes depending on the severity of astrogliosis, the presence of molecular markers such as iNOS and Ki67, and the ability of these cells to kill neighboring neurons through the accumulation of MAO-B-dependent H_2_O_2_. Mild reactive astrocytes contain increased MAO-B and GABA but not enough H_2_O_2_ to activate iNOS, lack Ki67, and show no signs of neuronal death^17^. In the current study, we observed mildly reactive astrocytes in the CA1-hippocampus of CIA mice with slight hypertrophy and elevated MAO-B and GABA but an absence of neuronal death. These mildly reactive astrocytes were in contrast to the severely reactive astrocytes observed in the GiD and APP/PS1-GiD mouse models of Alzheimer’s disease (AD)^16,17^, in which the severely reactive astrocytes exhibited MAO-B-dependent H_2_O_2_ production, iNOS, and Ki67, in addition to increased MAO-B and GABA. Most importantly, severely reactive astrocytes are associated with neuronal death in neighboring cells. The difference between the two animal models of RA and AD might be due to the difference in factors that trigger reactive astrogliosis: amyloid β plaques induce severely reactive astrocytes in AD and IL-1β induces mildly reactive astrocytes in RA. To confirm this hypothesis, it may be helpful to examine the characteristics of reactive astrogliosis in the postmortem brains of patients with RA.

Notably, we demonstrated that long-term inhibition of MAO-B with the reversible MAO-B inhibitor KDS2010 significantly improved arthritis and cognitive impairment. Consistently, previous clinical case reports suggested that patients with depression who were undergoing treatment with nonselective and irreversible MAO inhibitors (MAOIs), such as tranylcypromine, phenelzine, and isocarboxazid, showed improvements in depression, headache, and arthritis^31,32^. However, none of these irreversible inhibitors are used in the clinic to treat RA patients, possibly because long-term treatment with irreversible MAOIs results in waning effects or compensatory side effects. In fact, we have recently demonstrated that long-term treatment with the irreversible MAO-B inhibitor selegiline activates the major GABA-synthesizing enzyme D-amino-acid oxidase (DAO), resulting in a relapse of aberrant GABA^27^. Long-term treatment with the newly developed reversible MAO-B inhibitor KDS2010 significantly relieves arthritis and cognitive impairment in CIA mice. Therefore, KDS2010 overcomes the drawbacks of pre-existing irreversible MAO-B inhibitors and is suitable for long-term application in RA.

In the present study, we report that MAO-B and its byproduct GABA are present in the synovium and FLSs of patients with RA. Apart from MAO-B in the central nervous system, peripheral MAO-B is expressed in the liver, skin fibroblasts, and immune cells^14,15,57^. However, there have been no reports on the role of peripheral MAO-B in RA. However, in this study, the synovium and TNF-α-stimulated FLSs obtained from RA patients showed aberrant MAO-B expression and activity, raising the possibility that substrates for MAO-B were increased for the enzymatic reaction. We have demonstrated an increase in the precursor substrate putrescine and the direct substrate N-acetyl-GABA for MAO-B. Consistently, several studies have shown that putrescine is increased in RA compared to OA, which is a less inflammatory form of arthritis, and correlates with the degree of joint inflammation and destruction^58–60^. These studies have demonstrated that increased putrescine might be deleterious, leading to global DNA hypomethylation in FLSs, but spermidine, a derivative of putrescine, has been described as an autophagy inducer that contributes to maintaining homeostasis^61^. We demonstrated that in CIA mice and TNF-α-induced FLSs have an MAO-B-dependent inflammatory response, which is significantly inhibited by KDS2010. Moreover, according to a previous study, FLSs in RA in the absence of TNF-α showed significant increases in pathogenic RA signaling, such as NF-κB signaling, compared to FLSs in non-RA individuals^62^. These results suggest that RA FLSs in the absence of TNF-α might have higher MAO-B than non-RA FLSs. In addition, we found evidence of activated autophagy in RA, as previously reported^38,39^. These results suggest the possibility that autophagy in RA activates MAO-B, which in turn exacerbates RA. Thus, blocking MAO-B with KDS2010 could stop the vicious cycle of RA exacerbation. Therefore, we propose that directly targeting MAO-B in FLSs is a good therapeutic strategy to alleviate ongoing joint inflammation in RA.

The pharmacological inhibition of MAO-B with KDS2010 improved not only cognitive impairment but also joint inflammation in our study. However, because KDS2010 was systemically administered, there are concerns about the possibility that inhibiting joint inflammation ameliorated cognitive impairment. This concern is not unreasonable, considering the recent report that depression-like behavior, pain, and elevated levels of TNF-α in the hippocampus are persistent, even after the resolution of peripheral inflammation or the administration of pain killers in an RA animal model^23^. This study suggests that the hippocampus shows persistent inflammation, which is independent of peripheral inflammation. To address this concern, we used short-term treatment of KDS2010 during severe joint inflammation (Supplementary Fig. 2), which was accompanied by persistent hippocampal inflammation, as observed in a previous study^23^. These results suggest that cognitive impairment is dependent on central MAO-B but not peripheral MAO-B. This possibility should be validated in the future by cell-type-specific gene manipulations of central and peripheral MAO-B in animal models of RA.

In conclusion, our study provides mechanistic insights into MAO-B-dependent joint inflammation and MAO-B-dependent astrocytic GABA, which is associated with cognitive impairment in RA. We provide a potent pharmacological tool, KDS2010, that targets both peripheral and central MAO-B to alleviate RA symptoms associated with joints and the brain. These mechanistic insights and pharmacological tools should prove useful in developing better treatments for RA.

## Supplementary information

Supplementary text

## Data Availability

The data in this paper and the materials are available from the corresponding author (C.J.L.) upon request.
