# Supplementary material for: Inhibiting peripheral and central MAO-B ameliorates joint inflammation and cognitive impairment in rheumatoid arthritis
Source: Exp Mol Med. 2022 Aug 18;54(8):1188–200. doi: 10.1038/s12276-022-00830-z (PMC9440195; doi:10.1038/s12276-022-00830-z)
Supplement: Supplementary file 1 — Supplementary text [file 12276_2022_830_MOESM1_ESM.docx]

Supplementary information for

Title: Inhibition of peripheral and central MAO-B ameliorates both joint inflammation and cognitive impairment in rheumatoid arthritis

Running title: Monoamine oxidase-B is critical for rheumatoid arthritis

Woojin Won^a,b^, Hyun-Ji Choi^c^, Ji-Young Yoo^c^, Daeun Kim^b^, Tai Young Kim^b^, YeonHa Ju^b^, Ki Duk Park^d,e,f^, Hyunbeom Lee^g^, Sang Youn Jung^h,^*, and C. Justin Lee^a,b,^*

^a^KU-KIST Graduate School of Converging Science and Technology, Korea University, 145 Anam-ro, Seongbuk-gu, Seoul, 02841, Republic of Korea
^b^Center for Cognition and Sociality, Institute for Basic Science (IBS), Daejeon 34126, Republic of Korea
^c^Department of Biotechnology, CHA University, Seongnam, 13488, Republic of Korea
^d^Convergence Research Center for Diagnosis, Treatment and Care System of Dementia, Korea Institute of Science and Technology (KIST), Seoul 02792, Republic of Korea
^e^Division of Bio-Medical Science &Technology, KIST School, Korea University of Science and Technology, Seoul 02792, Republic of Korea
^f^KHU-KIST Department of Converging Science and Technology, Kyung Hee University, Seoul 02447, Republic of Korea
^g^Center for Advanced Biomolecular Recognition, Korea Institute of Science and Technology, Seoul 02792, South Korea
^h^Division of Rheumatology, Department of Internal Medicine, CHA Bundang Medical Center, CHA University, Seongnam, 13496 Republic of Korea.

*Sang Youn Jung
Division of Rheumatology, Department of Internal Medicine, CHA Bundang Medical Center, CHA University, Seongnam, 13496 Republic of Korea.
e-mail: jungsy7597@cha.ac.kr

*C. Justin Lee
Center for Cognition and Sociality, Institute for Basic Science (IBS), Daejeon 34126, Republic of Korea
e-mail: [cjl@ibs.re.kr](mailto:cjl@ibs.re.kr); Tel: +82-42-878-9150; fax: +82-42-878-9151

**Supplementary Information text**

**Bulk RNA sequencing**

Total RNA was prepared as previously described (1). RNA-Seq analysis was performed using 1µg of total RNA. For isolation of pure mRNA from the total RNA, we used Dynabeads mRNA DIRECT Kit (Invitrogen). Followed, library preparation was done by NEBNext Ultra RNA Library Prep Kit for Illumina (NEB). The quality of the library was evaluated using Agilent High Sensitivity DNA Kit (Agilent Technologies), and concentration was measured by Qubit 1X dsDNA HS Assay Kit (Invitrogen). 7 libraries (four control, and three TNF-α) were sequenced on an Illumina HiSeq 2500 on Rapid flow cells using HiSeq Rapid SBS Kit. Sequencing reads were aligned to human genome (hg38) using STAR aligner (2.7.8a) within Partek Flow pipeline. Quantification of gene counts were performed by Quantify to annotation model and number of gene reads was normalized with Median ratio (DESeq2). The reads were filtered by a false discovery rate (FDR) set-up p ≤ 0.05, and fold change ≥ ± 2. The reads were analyzed for the KEGG pathway using Partek flow pathway analysis (Build version 10.0.21.0929).

**Immunostaining of RA samples**

Primary FLS were cultured in a 24-well plate were stimulated with a TNF-α concentration-dependent manner (10, 20, 50 ng/ml). After 24 hours, FLS were fixed in 4% PFA and 0.5% glutaraldehyde for 15 min at RT, and washed in 0.1M PBS three times. The FLS were incubated for 1.5 hours in a blocking solution (0.3% Triton X-100; Sigma, 10% donkey serum in PBS) and then immunostained with a guinea-pig anti-GABA antibody (1:500) and goat anti-MAO-B antibody (1:500) overnight at 4 °C. After washing with PBS three times, cells were incubated with corresponding fluorescent secondary antibodies for 1.5 hours at RT and then rinses with PBS three times. During the second PBS washing step, DAPI staining was done by adding DAPI solution (1:3,000; Pierce). Coverslips were mounted on Polysine microscopic glass slides (Thermo Scientific). Images were acquired using a Nikon A1R confocal microscope.

Synovial tissue sections were post-fixed overnight in 4% paraformaldehyde at 4 °C and immersed in 30% sucrose for 48 hours for cryoprotection. Synovial tissue was cut into 8 μm-thick sections using a cryostat (Leica Microsystems Ltd). The samples were fixed with 2% formaldehyde and permeabilized by triton X-100 (Amresco) and incubated for 1 hour in a blocking solution (0.3% Triton-X, 5% normal goat serum in 0.1 M PBS; Cell signaling) and then immunostained with a mixture of primary antibodies including guinea-pig anti-GABA antibody (1:500) and goat anti-MAO-B (1:500) overnight at 4 °C. The slides were incubated with corresponding fluorescent secondary antibodies for 2 hours. A series of fluorescent images were obtained with a Zeiss LSM 510 META system (Carl Zeiss). 30-mm Z stack images in 5-mm steps were processed for further analysis using Zen software (Carl Zeiss).

**Image quantification**

Confocal microscopic images were analyzed using the ImageJ program. To quantify the expression of MAO-B and GABA in the synovium and FLS cells, the mean intensity value of MAO-B and GABA-positive pixels were measured. Every image was converted to 8-bit image, then MAO-B and GABA-positive binary image was defined.

**Western blot**

In case of cultured FLS, FLS were incubation with recombinant human TNF-α in a dose-dependent manner (10, 20, 50 ng/ml) at 37°C in 5% CO_2_ for 24 hours. Then, FLS were collected and lysed, as described in the above. Primary antibodies were incubated with the following primary antibodies in TNF-α-stimulated FLS: Beclin-1 (1:1000); Atg7 (1:000); LC3B-I (1:000); LC3B-II (1:1000); β-actin (1:1000). β-actin served as a loading control. Synovial tissues from OA and RA patients were lysed with RIPA buffer containing protease inhibitor cocktail for 30 min at 4°C. The protein concentrations were determined via a BCA kit. A total of 20 µg of protein were boiled in the presence of SDS sample buffer, then transferred onto a polyvinylidene fluoride microporous membrane (Millipore). The membrane was blocked with 5% skin milk for 1.5 hours at RT and incubated with the following primary antibodies at 4 °C for 24 hours: MAO-B (1:1000); LC3B-I (1:1000); LC3B-II (1:1000); GAPDH (1:1000). GAPDH served as a loading control. After washing three times with Tris-buffered saline containing 0.05% Tween 20, the blots were incubated with the corresponding horseradish peroxidase-conjugated secondary antibodies at RT for 2 hours. After incubation of secondary antibodies, the blots were developed by Immobilon Western ECL solution. The band intensity was acquired by ImageQunat LAS500 (GE Healthcare) and quantified using ImageJ program. Paw tissues from control, CIA, and CIA+KDS2010 were also prepared, as described in the above. Then, primary antibodies were incubated with the following primary antibodies: Cox-2 (1:1000); Beclin-1 (1:1000); ATG5 (1:1000); LC3B-I (1:1000); LC3B-II (1:1000); GAPDH (1:1000). In case of cultured FLS, FLS were incubation with recombinant human TNF-α in a dose-dependent manner (10, 20, 50 ng/ml) at 37°C in 5% CO_2_ for 24 hours.

**Intracellular H_2_O_2_ detection**

Intracellular H_2_O_2_ levels of FLS were determined using 2′,7′-dichlorodihydro-fluorescein diacetate (H_2_-DCFDA). FLS were stimulated with TNF-α (50 ng/ml) for 24 hours at 37°C in 5% CO_2_. Then these cells were washed with Hanks' Balanced Salt Solution (HBSS) containing Ca^2+^ and Mg^2+^, followed by application of 10 µM H_2_-DCFDA in HBSS containing Ca^2+^ and Mg^2+^ and incubated at 37°C for 20 min. Mean intensity of fluorescence was measured using multi-mode microplate readers (Molecular Devices) with an excitation wavelength of 485nm and an emission wavelength of 525 nm.

**Enzyme-linked immunosorbent assay and Luminex cytokine detection**

Measurement of proinflammatory cytokines (TNF-α and IL-6) in FLS and lysates of paw tissue were determined by Enzyme-linked immunosorbent assay (ELISA) kit (Thermofisher) according to the manufacturer’s protocol. Measurement of proinflammatory cytokines in serum, synovial fluid, and tissue supernatant from RA and OA patients were determined by Human MILLIPLEX kit (Millipore) on the MAGPIX-Luminex (Millipore) according to the manufacturer’s protocol. The kit includes premixed beads for 6 cytokines: IFN-γ, IL-10, IL-13, IL-1β, and IL-6, and TNF-α. Standard curves for each cytokine were prepared by serial dilution and run in duplicate. A MAGPIX-Luminex equipped with xPONENT v. 3.1 software was used to perform the multiplexed assays.

**ESI-LC-MS/MS-based metabolite quantification**

Metabolite analysis for putrescine, N-acetyl-GABA, and GABA was performed using EIS-LC-MS/MS. Exion LC AD UPLC coupling an MS/MS (Triple Quad 4500 System, AB Sciex LLC) using an Acquity UPLC BEH HILIC column (1.7 ethyl-GABA, and GA 2.1 mm x 100 mm, Waters) at 30°C, controlled by Analyst software (AB Sciex LP). 70% methanol (100 µl) was added and vortexed with the FLS sample pellets. Cells were lysed by three consecutive freeze/thaw cycles using liquid nitrogen, and the lysate was centrifuged for 10 min at 14,000 rpm. 20 ml of the supernatant from each sample was added with 5 ml of internal standard (d2-GABA at a final concentration of 4 µM) and vortexed for 30 s. The mixture was evaporated to dryness at 37°C under a nitrogen. The residue was reconstituted with 25 ml of the mobile phase A (0.1% formic acid in acetonitrile): B (50mM ammonium formate, pH 4) = 8:2 solvents by vortexing for 30 s, and sonicating for 15 min. The initial chromatographic conditions were 80% of solvent A at a flow rate of 0.4 ml·min-1. After 7 min at 20% of solvent B, it was set to 95% over the next 30 s, and these conditions were retained for an additional 1 min. The system was then returned to the initial conditions over the next 30 s. The system was re-equilibrated in the initial conditions. The total running time was 10.5 min. All samples were maintained at 7°C during the analysis, and the injection volume was 5 ml. The MS analysis was performed using ESI in positive mode. The vaporizer temperature and ion spray voltage were 380°C and 5.5 kV, respectively. The curtain gas was maintained at 35 psi, and the collision gas was kept at 8 psi. The nebulizer gas was 60 psi, and turbo gas flow rate was 70 psi. The metabolites were detected selectively using their unique multiple reaction monitoring (MRM) pairs. The following MRM mode (Q1 / Q3) was selected: putrescine (m/z 89 to m/z 72), N-acetyl-GABA (m/z 146 to m/z 86) and GABA (m/z 103 to m/z 87) to monitor specific parent-to-product transitions. The standard calibration curve for each metabolite was performed for quantification.

**Histological analysis of mice joint tissue**

Mice hind paws from each group were fixed in 4% PFA, decalcified with 0.5 M EDTA at pH 8.0 for 4 weeks, and was subsequently dehydrated and embedded in paraffin. The tissue sections were cut at 4–6 μm thickness in a sagittal orientation and stained with hematoxylin and eosin (H&E), then the stained sections of ankle joints from each mouse were evaluated for pannus formation, inflammatory infiltrate, and bone erosion based on previously described (2).

**Novel object recognition**

To examine recognition memory, mice were habituated in to an open field (40 x 40 cm square with 40 cm high walls). After habituation, mice were placed in to the open field with two identical objects positioned in the middle of first and the second quadrant of the cage. Simple visual cue was placed on the walls to provide spatial reference for mice. Mice were allowed to explore these objects for 10 min and returned to the home cage for one hour while one of the familiar objects was replaced with a novel object. Then, mice were exposed for 10 min to a novel object. The DI was calculated as the percentage of time spent on the novel object over the total time spent on both novel and familiar object.

**Novel place recognition**

To investigate spatial recognition memory, novel place recognition was employed. Basic principle was same with the novel object recognition, but one of the two identical objects was placed at a novel place, opposite quadrant of the open field. Thus, the DI was calculated as the percentage of time spent on the novel place over the total time spent on both novel and familiar place.

**Slice immunostaining for confocal microscopy**

Mice were deeply anesthetized by 2% avertin intraperitoneally (i.p.) and perfused with 0.9% saline followed by 4% paraformaldehyde (PFA). Brains were excised and postfixed overnight at 4ºC in 4% PFA, then immersed in 30% sucrose for 48 hours for cryoprotection. Coronal hippocampal sections were cut at 30-µm-thickness and stored in storage solution at 4ºC. Sections were washed in 0.1 M PBS three times, and incubated for one hour in a blocking solution (0.3% Triton X-100, 4% donkey serum in PBS) in RT followed by immunostained with primary antibodies on a shaker at 4ºC overnight. After washing in PBS three times, sections were incubated with corresponding fluorescent secondary antibodies for one-hour at RT and then washed with PBS. During the second washing step, DAPI staining was done by adding DAPI solution (1: 2,000; Pierce). Lastly, immunostained sections were mounted on coverslide with fluorescent mounting medium (Dako). A series of fluorescent images were obtained with a Nikon A1 confocal microscope, 24-µm Z stack images in 2-µm steps were processed for Sholl analysis using the ImageJ (NIH) program. Primary antibodies were diluted to the following amounts: chicken anti-GFAP (1:500); and guinea pig anti-GABA (1:200). Secondary antibodies were diluted (1:500) in the blocking solution. In case of proinflammatory cytokines incubation conditions, slices were incubated with IL-1β (20 ng/ml), and KDS2010 (1 µM) at least two-hour before recording, respectively.

**Image quantification**

Confocal microscopic images were analyzed using the ImageJ program. To quantify astrocytic GABA contents, the mean intensity value of GABA-positive pixels in the GFAP-positive were measured. Every image was converted to 8-bit image, then GFAP and GABA-positive binary image was defined. Then, GFAP-positive binary image and GABA-positive image were multiplied, which showed remaining GABA-positive signal only in the GFAP-positive pixels. For measuring GFAP-positive area, GFAP-positive image was transformed into binary and the white pixels were measured. Next, to evaluate the reactivity of astrocytes, Sholl analysis, provided by ImageJ program, was applied. We performed Sholl anlaysis with 8-bit GFAP-positive binary images and it draws serial circle at 10-µm intervals from the center of the DAPI signal. Ramification index and the number of intercepts of GFAP processes were automatically measured from the starting radius.

**Assessment of MAO-B enzyme activity**

Using commercially available kit for MAO-B enzyme activity assay (Thermo Fisher Scientific), MAO-B activity in the hippocampus of CIA mice was examined. Lysates of the hippocampal tissue and paw tissue were prepared by centrifuging at 16,000rpm after homogenization with protease inhibitor cocktail and RIPA lysis buffer. Protein concentration in the supernatant from the lysates was quantified with a Bradford-based assay. Then, equal amounts of samples were pre-incubated for 30 min at RT and added to individual wells of a 96-well microplate. The fluorometric assay was performed with the mixture of Amplex Red, horseradish peroxidase, and benzyl amine which is a substrate of MAO-B. After 30 min incubation, fluorescence was measured by multi-mode microplate readers (Molecular Devices).

**Quantitative real-time PCR**

Quantitative RT-PCR was performed by using SYBR Green PCR Master Mix. Reactions were performed in triplicates in a total volume of 10 µL containing 4 µL cDNA, 10 pM primers, and 5 µL power SYBR Green PCR Master Mix (Applied Biosystems). The mRNA level of *Mao-b* was normalized to that of that of *Gapdh* mRNA, then fold-induction was calculated using the 2^-ΔΔCT^ method.

**Electrophysiology**

Mice were deeply anaesthetized with 3% isoflurane followed by decapitation. Then, the brain was excised from the skull soon as possible, and submerged in chilled cutting solution that contained (in mM): 250 of sucrose; 26 of NaHCO_3_; 10 of d(+)-glucose; 4 of MgCl_2_; 3 of myo-inositol; 2.5 of KCl; 2 of sodium pyruvate; 1.25 of NaH_2_PO_4_; 0.5 of ascorbic acid; 0.1 of CaCl2_;_ and 1 of kynurenic acid, pH 7.4.. After trimming the brain, 300 µm-thick transverse hippocampal slices were cut using a vibrating microtome (PRO7N; DSK) and transferred to an artificial cerebrospinal fluid (aCSF) solution (in mM): 130 of NaCl, 24 of NaHCO_3_, 1.25 of NaH_2_PO_4_, 3.5 KCl, 1.5 of CaCl_2_, 1.5 of MgCl_2_, and 10 of d(+)-glucose, pH 7.4. All solutions were gassed with 95% O_2_ and 5% CO_2_. Slices were incubated at RT for at least one-hour before recording. In case of proinflammatory cytokines incubation conditions, slices were incubated with IL-1β (20 ng/ml), TNF-α (100 ng/ml), or IL-6 (10 ng/ml) at least two-hour before recording, respectively.

For recording of tonic GABA currents, slices were transferred to a recording slice chamber which were mounted on an upright Zeiss microscope and continuously perfused with the aCSF solution. CA1-hippocampal pyramidal neurons were visualized by a 60x water immersion objective (numerical aperture = 0.90) with infrared differential interference contrast optics, a CMOS camera (Hamamatsu Photonics) and the Imaging Workbench software (INDEC BioSystems). Whole-cell-patch recordings were performed from CA1-hippocampal pyramidal neuron. The holding potential was -60 mV. Pipette resistance was typically 6–8 MΩ and the pipette was filled with an internal solution consisting of (in mM): 135 of CsCl; 4 of NaCl; 0.5 of CaCl2; 10 of HEPES; 5 of EGTA; 2 of Mg-ATP; 0.5 of Na2-GTP; and 10 of QX-314, pH-adjusted to 7.2 with CsOH (278–285 mOsmol). Before measuring the tonic current, the baseline was stabilized with D-AP5 (50 μM) and CNQX (20 μM). Electrical signals were digitized and sampled at 10-ms intervals with Digidata 1550B data acquisition system and the Multiclamp 700B Amplifier (Molecular Devices) using the pClamp10.2 software. For investigating the change of extrasynaptic GABA receptors, tonic current was measured by the baseline shift between before and after bicuculline (50 μM) administration under saturating concentration of GABA (10 μM) treatment. The frequency and amplitude of spontaneous inhibitory postsynaptic currents before bicuculline administration was detected and measured by Mini Analysis (Synaptosoft).

**Injection of IL-1 receptor antagonist**

CIA mice were injected with IL-1 receptor antagonist (IL-1ra, recombinant Mouse IL-1ra/IL-F3 protein, R&D) in CIA mice to neutralize the IL-1β activity. Total of 5 µg in 100 µl of IL-1ra was subcutaneously (s.c.) injected for four consecutive days in CIA mice.

**Guide Cannulae implantation and infusion of IL-1β**

Mice were deeply anesthetized with vaporized 1% isoflurane and immobilized in a stereotaxic frame (RWD Life Science, China). Following an incision on the midline of the scalp, bilateral craniotomies were performed using a microdrill. Guide cannulae were bilaterally implanted into the hippocampus CA1 (AP, -1.7 mm; ML, ±1.2 mm; DV, -1.15 mm from the bregma). A total of 20ng in 1 µl of IL-1β (R&D) was injected at a rate of 0.2 µl/min, which was precisely controlled by the syringe pump (KD Scientific, MA, USA). Then an additional 5 min was allowed for protein diffusion within the brain and then withdrawn. The procedures of injection were implemented prior to 2 weeks of behaviors. Artificial cerebrospinal fluid (aCSF) was used for control.

**Supplementary References**

1 Crist, A. M. *et al.* Transcriptomic analysis to identify genes associated with selective hippocampal vulnerability in Alzheimer's disease. *Nat. Commun.* **12**, 2311 (2021).

2 Jung, S. Y. *et al.* Synergistic Effect of Tolerogenic Dendritic Cells and Etanercept on a Collagen-induced Arthritis Animal Model. *J. Hard Tissue Biol.* **28**, 265-271 (2019).

**Supplementary figures**


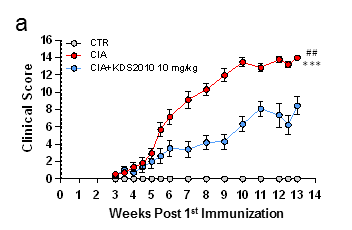


Supplementary Fig. 1. KDS2010 (10 mg/kg/day) alleviates severity of RA. *** P < 0.001 for CTR vs. CIA; ## P < 0.01 for CIA vs. CIA+KDS2010 (10 mg/kg/day). Error bars in the graphs indicate standard errors of mean. Statistical details are provided in Supplementary Table 1.


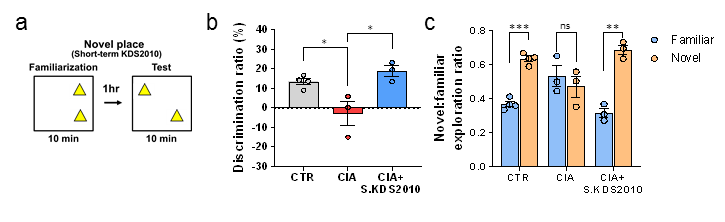


Supplementary Fig. 2. Short-term treatment of KDS2010 rescue cognitive impairment. (a) The schematic images of NPR test. (b, c) The result of NPR test with a short-term treatment of KDS2010 (10 mg/kg/day) in CIA+KDS2010 mice (two-tailed paired t-test, n = 4, 3, and 3). Error bars in the graphs indicate SEM. * P < 0.05; ** P < 0.01; *** P < 0.001; ns, non-significant. Statistical details are provided in Supplementary Table 1.


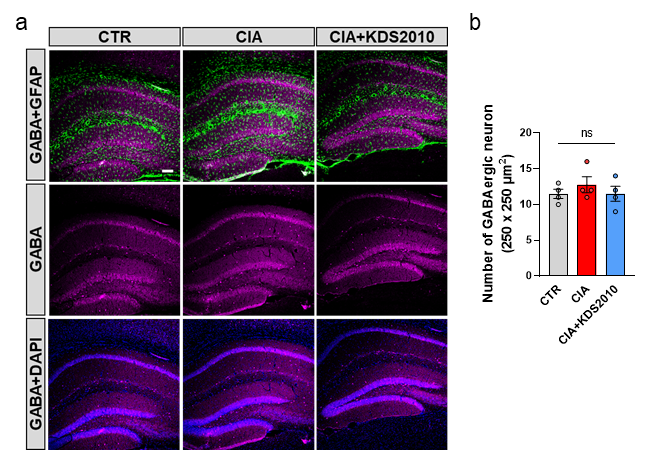


Supplementary Fig. 3. No difference of the number of GABA cells in CA1-hippocampus. (a) Representative confocal image of GABA, GFAP, and DAPI with low-magnified images. (b) Comparison of the number of GABAergic neuron in CA1-hippocampus.


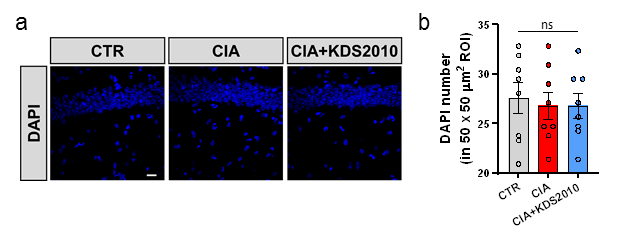


Supplementary Fig. 4. No sign of cell death based on DAPI-positive pyramidal neuron in CA1-hippocampus. (a) Representative confocal images illustrating the DAPI signals of CA1-hippocampus (Sale bar, 10µm). (b) Number of DAPI signals in 50 × 50 µm^2^ of the CA1-hipocampus of the control, CIA and CIA+KDS2010 mice (n = 8 for each groups). Error bars in the graphs indicate SEM. ns, non-significant. (one-way ANOVA with Tukey's multiple comparisons test). Statistical details are provided in Supplementary Table 1.


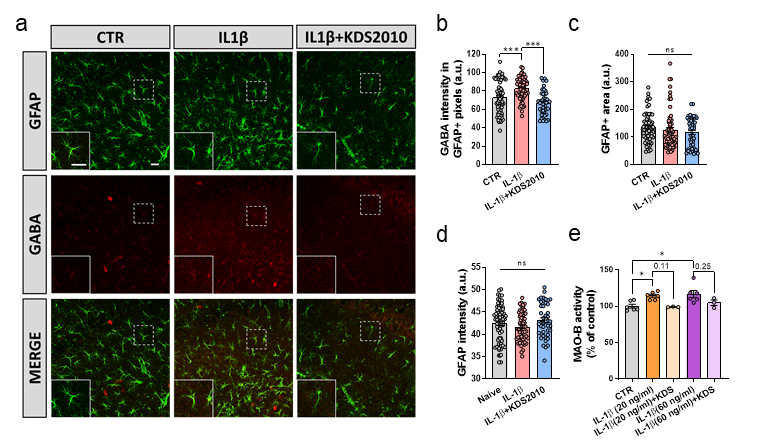


Supplementary Fig. 5. IL-1β stimulates MAO-B-dependent hippocampus astrocytic GABA production. (a) Representative confocal images illustrating the GFAP and GABA in the CA1-hippocampus of IL-1β (20 ng/ml) and KDS2010 (1 µM) incubations ex vivo (Sale bar, 10µm). (b) GABA intensity in GFAP-positive pixel of the incubated brain (Kruskal-Wallis test with uncorrected Dunn's multiple comparisons test, Naïve, n=69; IL-1β, n=60; IL-1β+KDS2010, n=44). (c) Summary graph of GFAP-positive area (Kruskal-Wallis test with uncorrected Dunn's multiple comparisons test). (d) Summary graph of GFAP-positive intensity (one-way ANOVA with Tukey's multiple comparisons test). (e) MAO-B enzyme assay with IL-1β (20 and 60 ng/ml) and KDS2010-treated hippocampal astrocyte culture. Error bars in the graphs indicate SEM. *** P < 0.001; ns, non-significant. Statistical details are provided in Supplementary Table 1.


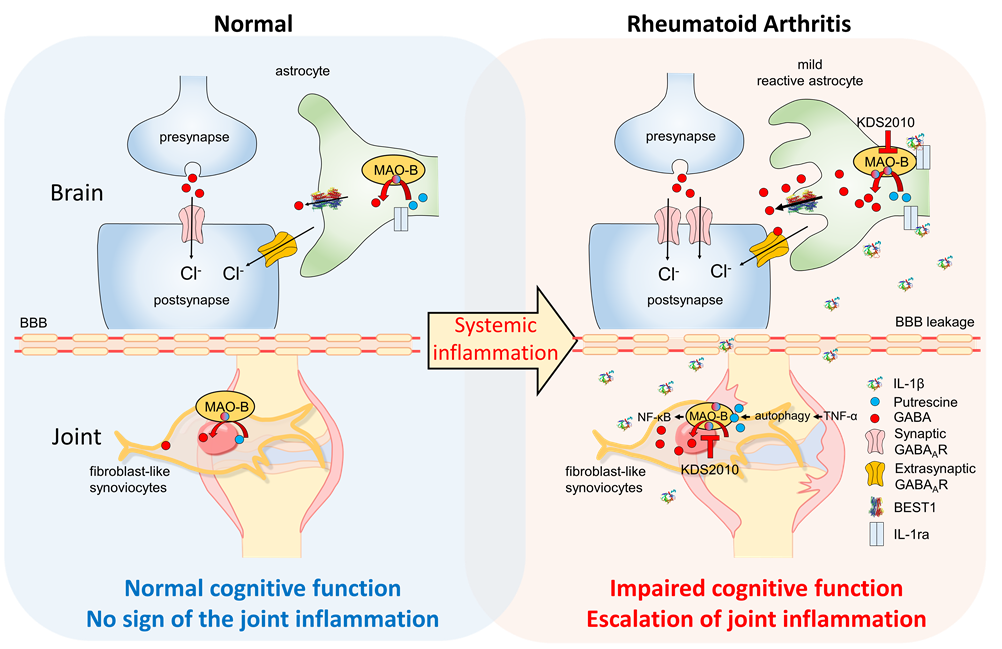


Supplementary Fig. 6. Schematic diagram of mechanism of MAO-B-dependent joint inflammation and cognitive impairment and in RA. In normal case, both FLS cells and hippocampal astrocytes shows low expression level of MAO-B. In contrast, when rheumatoid arthritis followed by systemic inflammation, MAO-B and its activity is upregulated in the joint tissue and brain. Thus activation of MAO-B in RA might escalate the joint inflammation through its products and MAO-B induces cognitive impairment via astrocyte tonic GABA. However, reversible MAO-B inhibitor which is KDS2010, rescues MAO-B-dependent joint inflammation and MAO-B-dependent astrocytic GABA associated with cognitive impairment in RA.

Supplementary Table 1. Detail statistical analysis

| **Fig. No.** | **Results from statistical analysis** |
| --- | --- |
| Fig. 2b | Kruskal-Wallis test with uncorrected Dunn's multiple comparisons test |
|  | CTR (n=29) vs. TNF-α 10 (ng/ml) (n=24), p<0.001; CTR vs. TNF-α 20 (ng/ml) (n=22), p<0.001; CTR vs. TNF-α 50 (ng/ml) (n=55), p<0.001; TNF-α 10 (ng/ml) vs. TNF-α 20 (ng/ml), p>0.99; TNF-α 10 (ng/ml) vs. TNF-α 50 (ng/ml), p=0.01; TNF-α 20 (ng/ml) vs. TNF-α 50 (ng/ml), p=0.73 |
| Fig. 2c | Kruskal-Wallis test with uncorrected Dunn's multiple comparisons test |
|  | CTR (n=29) vs. TNF-α 10 (ng/ml) (n=24), p<0.001; CTR vs. TNF-α 20 (ng/ml) (n=22), p<0.04; CTR vs. TNF-α 50 (ng/ml) (n=55), p<0.001; TNF-α 10 (ng/ml) vs. TNF-α 20 (ng/ml), p=0.34; TNF-α 10 (ng/ml) vs. TNF-α 50 (ng/ml), p=0.02; TNF-α 20 (ng/ml) vs. TNF-α 50 (ng/ml), p<0.001 |
| Fig. 2f | unpaired t-test, two-tailed |
|  | CTR (n=43) vs. TNF-α (n=43), p<0.001, t=6.709, df=84 |
| Fig. 2g | unpaired t-test, two-tailed |
|  | CTR (n=2) vs. TNF-α (n=2), p=0.03, t=6.146, df=2 |
| Fig. 2i | Mann Whitney test, two-tailed |
|  | CTR (n=6) vs. TNF-α (n=6); U=0, p=0.002 |
| Fig. 2j | Mann Whitney test, two-tailed |
|  | CTR (n=6) vs. TNF-α (n=6); U=5, p=0.04 |
| Fig. 2k | Mann Whitney test, two-tailed |
|  | CTR (n=6) vs. TNF-α (n=6); U=8, p=0.13 |
| Fig. 2l | one-way ANOVA with Tukey's multiple comparisons test |
|  | IFN-γ, Noraml (n=20) vs. OA (n=16), p=0.004; Normal vs. RA (n=16), p<0.001; OA vs. RA, p<0.001; F (2,49)=31.48, p<0.001; IL-10, Noraml (n=20) vs. OA (n=16), p<0.004; Normal vs. RA (n=16), p=0.28; OA vs. RA, p=0.003; F (2,49)=14.15, p<0.001; IL-13, Normal (n=20) vs. OA (n=16), p<0.001; Normal vs. RA (n=16), p<0.001; OA vs. RA, p<0.001; F (2,49)=3042, p<0.001; IL-1β, Normal (n=20) vs. OA (n=16), p=0.90; Normal vs. RA (n=16), p<0.001; OA vs. RA, p<0.001; F (2,49)=30.65, p<0.001; IL-6, Normal (n=20) vs. OA (n=16), p=0.94; Normal vs. RA (n=16), p<0.001; OA vs. RA, p<0.001; F (2,49)=43.90, p<0.001; TNF-α, Normal (n=20) vs. OA (n=16), p<0.001; Normal vs. RA (n=16), p<0.001; OA vs. RA, p=0.96; F (2,49)=20.22, p<0.001 |
| Fig. 2m | unpaired t-test, two-tailed |
|  | IFN-γ, OA synovial fluid (n=12) vs. RA synovial fluid (n=16), p=0.02, t=2.455, df=18.53; IL-10, OA synovial fluid (n=15) vs. RA synovial fluid (n=16), p=0.005, t=3.013, df=29.00; IL-13, OA synovial fluid (n=15) vs. RA synovial fluid (n=16), p=0.22, t=1.260, df=29.00; IL-1β, OA synovial fluid (n=14) vs. RA synovial fluid (n=16), p=0.02, t=2.701, df=16.33; IL-6, OA synovial fluid (n=16) vs. RA synovial fluid (n=16), p=0.005, t=3.027, df=28.94; TNF-α, OA synovial fluid (n=15) vs. RA synovial fluid (n=16), p=0.05, t=2.097, df=22.84 |
| Fig. 2n | unpaired t-test, two-tailed |
|  | IFN-γ, OA tissue (n=4) vs. RA tissue (n=6), p=0.28, t=1.156, df=8.00; IL-10, OA tissue (n=4) vs. RA tissue (n=6), p=0.16, t=1.560, df=8.00; IL-13, OA tissue (n=4) vs. RA tissue (n=6), p=0.36, t=0.9788, df=8.00; IL-1β, OA tissue (n=4) vs. RA tissue (n=6), p=0.19, t=1.436, df=8.00; IL-6, OA tissue (n=4) vs. RA tissue (n=6), p=0.33, t=1.034, df=8.00; TNF-α, OA tissue (n=4) vs. RA tissue (n=6), p=0.28, t=1.146, df=8.00, assumed normal distribution |
| Fig. 2o | paired t-test, two-tailed |
|  | OA (n=2) vs. RA (n=2), p=0.02, t=8.067, df=2 |
| Fig. 2q | unpaired t-test, two-tailed |
|  | OA (n=3) vs. RA (n=3), p<0.001, t=52.53, df=4 |
| Fig. 2r | unpaired t-test, two-tailed |
|  | OA (n=3) vs. RA (n=3), p=0.008, t=4.825, df=4 |
| Fig. 2s | unpaired t-test, two-tailed |
|  | OA (n=3) vs. RA (n=3), p=0.20, t=1.527, df=4 |
| Fig. 3b | two-way ANOVA with Tukey's multiple comparisons test |
|  | CTR (n=2) vs. CIA (n=7), p<0.001; CTR vs. CIA+KDS2010 10 mpk (n=5), p=0.005; CTR vs. CIA+KDS2010 30mpk (n=4), p=0.08, CIA vs. CIA+KDS2010 10mpk, p<0.001; CIA vs. CIA+KDS2010 30mpk, p<0.001; CIA+KDS2010 10mpk vs. CIA+KDS2010 30mpk, p=0.30; effect of time and group F(57, 266)=5.408, p<0.001 |
| Fig. 3c | two-way ANOVA with Tukey's multiple comparisons test |
|  | CTR (n=2) vs. CIA (n=7), p<0.001; CTR vs. CIA+KDS2010 10 mpk (n=5), p=0.01; CTR vs. CIA+KDS2010 30mpk (n=4), p=0.74, CIA vs. CIA+KDS2010 10mpk, p<0.001; CIA vs. CIA+KDS2010 30mpk, p<0.001; CIA+KDS2010 10mpk vs. CIA+KDS2010 30mpk, p=0.06; effect of time and group F(57, 280)=1.879, p<0.001 |
| Fig. 3e | one-way ANOVA with Tukey's multiple comparisons test |
|  | CTR (n=3) vs. CIA (n=3), p<0.001; CTR vs. CIA+KDS2010 10 mpk (n=3), p=0.55; CTR vs. CIA+KDS2010 30mpk (n=3), p=0.46, CIA vs. CIA+KDS2010 10mpk, p=0.003; CIA vs. CIA+KDS2010 30mpk, p=0.003; CIA+KDS2010 10mpk vs. CIA+KDS2010 30mpk, p>0.99; F (3,8)=18.29, p<0.001 |
| Fig. 3f | one-way ANOVA with Tukey's multiple comparisons test |
|  | CTR (n=3) vs. CIA (n=3), p<0.001; CTR vs. CIA+KDS2010 10 mpk (n=3), p=0.26; CTR vs. CIA+KDS2010 30mpk (n=3), p=0.34, CIA vs. CIA+KDS2010 10mpk, p=0.001; CIA vs. CIA+KDS2010 30mpk, p<0.001; CIA+KDS2010 10mpk vs. CIA+KDS2010 30mpk, p>0.99; F (3,8)=25.65, p<0.001 |
| Fig. 3g | one-way ANOVA with Tukey's multiple comparisons test |
|  | CTR (n=3) vs. CIA (n=3), p<0.001; CTR vs. CIA+KDS2010 10 mpk (n=3), p=0.19; CTR vs. CIA+KDS2010 30mpk (n=3), p=0.47, CIA vs. CIA+KDS2010 10mpk, p=0.01; CIA vs. CIA+KDS2010 30mpk, p=0.005; CIA+KDS2010 10mpk vs. CIA+KDS2010 30mpk, p=0.87; F (3,8)=15.12, p<0.001 |
| Fig. 3h | one-way ANOVA with Tukey's multiple comparisons test |
|  | CTR (n=4) vs. CIA (n=6), p<0.001; CTR vs. CIA+KDS2010 (n=3), p=0.35; CIA vs. CIA+KDS2010, p<0.001; F (2,24)=37.02, p<0.001 |
| Fig. 3j | one-way ANOVA with Tukey's multiple comparisons test |
|  | CTR (n=4) vs. CIA (n=5), p<0.001; CTR vs. CIA+KDS2010 (n=4), p=0.08; CIA vs. CIA+KDS2010, p=0.003; F (2,10)=26.24, p<0.001 |
| Fig. 3k | one-way ANOVA with Tukey's multiple comparisons test |
|  | CTR (n=2) vs. CIA (n=3), p=0.02; CTR vs. CIA+KDS2010 (n=2), p=0.03; CIA vs. CIA+KDS2010, p=0.92; F (2,4)=12.55, p=0.02 |
| Fig. 3l | one-way ANOVA with Tukey's multiple comparisons test |
|  | CTR (n=2) vs. CIA (n=3), p=0.22; CTR vs. CIA+KDS2010 (n=2), p=0.05; CIA vs. CIA+KDS2010, p=0.009; F (2,4)=17.91, p=0.01 |
| Fig. 3m | one-way ANOVA with Tukey's multiple comparisons test |
|  | CTR (n=2) vs. CIA (n=3), p=0.37; CTR vs. CIA+KDS2010 (n=2), p=0.97; CIA vs. CIA+KDS2010, p=0.29; F (2,4)=1.967, p=0.25 |
| Fig. 3o | one-way ANOVA with Tukey's multiple comparisons test |
|  | CTR (n=75) vs. CIA (n=58), p<0.001; CTR vs. CIA+KDS2010 10 mpk (n=52), p=0.36; CTR vs. CIA+KDS2010 30mpk (n=52), p=0.77, CIA vs. CIA+KDS2010 10mpk, p<<0.001; CIA vs. CIA+KDS2010 30mpk, p<0.001; CIA+KDS2010 10mpk vs. CIA+KDS2010 30mpk, p=0.92; F (3,233)=39.01, p<0.001 |
| Fig. 3p | one-way ANOVA with Tukey's multiple comparisons test |
|  | CTR (n=75) vs. CIA (n=58), p<0.001; CTR vs. CIA+KDS2010 10 mpk (n=52), p<0.001; CTR vs. CIA+KDS2010 30mpk (n=52), p<0.001, CIA vs. CIA+KDS2010 10mpk, p<<0.001; CIA vs. CIA+KDS2010 30mpk, p<0.001; CIA+KDS2010 10mpk vs. CIA+KDS2010 30mpk, p<0.001; F (3,233)=210.9, p<0.001 |
| Fig. 3s | one-way ANOVA with Tukey's multiple comparisons test |
|  | CTR (n=4) vs. CIA (n=6), p<0.001; CTR vs. CIA+KDS2010 (n=4), p=0.08; CIA vs. CIA+KDS2010, p=0.004; F (2,11)=24.67, p<0.001 |
| Fig. 4b | one-way ANOVA with Tukey's multiple comparisons test |
|  | CTR (n=14) vs. CIA (n=18), p<0.001; CTR vs. CIA+KDS2010 (n=16), p>0.99; CIA vs. CIA+KDS2010, p<0.001; F (2,45)=16.95, p<0.001 |
| Fig. 4c | two-way ANOVA with Tukey's multiple comparisons test |
|  | CTR (n=14), Familiar vs. Novel, p<0.001; CIA (n=18), Familiar vs. Novel, p=0.05; CIA+KDS2010 10mpk (n=16), p<0.001, effect of group F(1, 45)=14.40, p<0.001 |
| Fig. 4e | one-way ANOVA with Tukey's multiple comparisons test |
|  | CTR (n=9) vs. CIA (n=10), p<0.001; CTR vs. CIA+KDS2010 (n=8), p=0.76; CIA vs. CIA+KDS2010, p<0.001; F (2,24)=28.89, p<0.001 |
| Fig. 4f | two-way ANOVA with Tukey's multiple comparisons test |
|  | CTR (n=9), Familiar vs. Novel, p<0.001; CIA (n=10), Familiar vs. Novel, p=0.34; CIA+KDS2010 10mpk (n=8), p<0.001, effect of group F(1, 24)=64.15, p<0.001 |
| Fig. 5c | one-way ANOVA with Tukey's multiple comparisons test |
|  | CTR (N=5, n=53) vs. CIA (N=6, n=104), p<0.001; CTR vs. CIA+KDS2010 (N=6, n=67), p=0.78; CIA vs. CIA+KDS2010, p<0.001; F (2,221)=55.92, p<0.001 |
| Fig. 5d | one-way ANOVA with Tukey's multiple comparisons test |
|  | CTR (N=3, n=46) vs. CIA (N=3 n=47), p=0.04; CTR vs. CIA+KDS2010 (N=3, n=41), p<0.001; CIA vs. CIA+KDS2010, p<0.01; F (2,131)=34.85, p<0.001 |
| Fig. 5e | one-way ANOVA with Tukey's multiple comparisons test |
|  | CTR (N=4) vs. CIA (N=4), p=0.06; CTR vs. CIA+KDS2010 (N=4), p=0.23; CIA vs. CIA+KDS2010, p=0.004; F (2,9)=10.22, p=0.005 |
| Fig. 5f | one-way ANOVA with Tukey's multiple comparisons test |
|  | CTR (N=2) vs. CIA (N=3), p=0.02; CTR vs. CIA+KDS2010 (N=2), p=0.57; CIA vs. CIA+KDS2010, p=0.41; F (2,4)=1.251, p=0.38, |
| Fig. 5g | Kruskal-Wallis test with uncorrected Dunn's multiple comparisons test |
|  | CTR (N=4, n=30) vs. CIA (N=4, n=30), p=0.02; CTR vs. CIA+KDS2010 (N=4, n=36), p>0.99; CIA vs. CIA+KDS2010, p=0.003 |
| Fig. 5h | one-way ANOVA with Tukey's multiple comparisons test |
|  | CTR (N=4, n=8) vs. CIA (N=4, n=9), p=0.002; CTR vs. CIA+KDS2010 (N=4, n=9), p=0.46; CIA vs. CIA+KDS2010, p=0.02; F (2,23)=8.405, p=0.002 |
| Fig. 5i | one-way ANOVA with Tukey's multiple comparisons test |
|  | CTR (N=3, n=7) vs. CIA (N=3, n=7), p=0.93; CTR vs. CIA+KDS2010 (N=3, n=7), p=0.95; CIA vs. CIA+KDS2010, p=0.79; F (2,18)=0.22, p=0.80 |
| Fig. 5k | one-way ANOVA with Tukey's multiple comparisons test |
|  | CTR (N=3, n=15) vs. CIA (N=3, n=23), p=0.03; CTR vs. CIA+KDS2010 (N=3, n=17), p=0.96; CIA vs. CIA+KDS2010, p=0.05; F (2,52)=4.518, p=0.02 |
| Fig. 5l | one-way ANOVA with Tukey's multiple comparisons test |
|  | CTR (N=3, n=15) vs. CIA (N=3, n=23), p=0.30; CTR vs. CIA+KDS2010 (N=3, n=17), p=0.96; CIA vs. CIA+KDS2010, p=0.43; F (2,52)=1.360, p=0.27 |
| Fig. 5n | one-way ANOVA with Tukey's multiple comparisons test |
|  | CTR (N=5, n=21) vs. CIA (N=6 n=21), p<0.001; CTR vs. CIA+KDS2010 (N=6, n=19), p<0.001; CIA vs. CIA+KDS2010, p>0.99; F (2,58)=13.33, p<0.001 |
| Fig. 5o | one-way ANOVA with Tukey's multiple comparisons test |
|  | CTR (N=3, n=8) vs. CIA (N=3, n=7), p=0.15; CTR vs. CIA+KDS2010 (N=4, n=9), p=0.10; CIA vs. CIA+KDS2010, p=0.77; F (2,21)=2.908, p=0.08 |
| Fig. 5p | one-way ANOVA with Tukey's multiple comparisons test |
|  | CTR (N=3, n=8) vs. CIA (N=3, n=7), p=0.03; CTR vs. CIA+KDS2010 (N=4, n=9), p>0.99; CIA vs. CIA+KDS2010, p=0.02; F (2,21)=5.227, p=0.01 |
| Fig. 5q | one-way ANOVA with Tukey's multiple comparisons test |
|  | CTR (N=5, n=21) vs. CIA (N=6, n=21), p<0.001; CTR vs. CIA+KDS2010 (N=6, n=19), p<0.001; CIA vs. CIA+KDS2010, p>0.99; F (2,58)=13.33, p<0.001 |
| Fig. 5r | one-way ANOVA with Tukey's multiple comparisons test |
|  | CTR (N=5, n=21) vs. CIA (N=6, n=21), p=0.74; CTR vs. CIA+KDS2010 (N=6, n=19), p=0.54; CIA vs. CIA+KDS2010, p=0.94; F (2,58)=0.6061, p=0.55 |
| Fig. 6b | Kruskal-Wallis test with uncorrected Dunn's multiple comparisons test |
|  | N=3 for each groups; Naïve (n=9) vs. IL-1β (n=9), p<0.001; Naïve vs. TNF-α (n=7), p>0.99; Naïve vs. IL-6 (n=7), p=0.83; IL-1β vs. TNF-α, p=0.09; IL-1β vs. IL-6, p=0.13; TNF-α vs. IL-6. p>0.99 |
| Fig. 6c | Kruskal-Wallis test with uncorrected Dunn's multiple comparisons test |
|  | N=3 for each groups; Naïve (n=9) vs. IL-1β (n=9), p<0.07; Naïve vs. TNF-α (n=7), p>=0.15; Naïve vs. IL-6 (n=7), p=0.46; IL-1β vs. TNF-α, p>0.99; IL-1β vs. IL-6, p>0.99; TNF-α vs. IL-6. p>0.99 |
| Fig. 6d | Kruskal-Wallis test with uncorrected Dunn's multiple comparisons test |
|  | N=3 for each groups; Naïve (n=9) vs. IL-1β (n=9), p=0.03; Naïve vs. TNF-α (n=7), p>0.99; Naïve vs. IL-6 (n=7), p>0.99; IL-1β vs. TNF-α, p=0.005; IL-1β vs. IL-6, p=0.14; TNF-α vs. IL-6. p>0.99 |
| Fig. 6e | Kruskal-Wallis test with uncorrected Dunn's multiple comparisons test |
|  | N=3 for each groups; Naïve (n=9) vs. IL-1β (n=10), p>0.99; Naïve vs. TNF-α (n=7), p>0.99; Naïve vs. IL-6 (n=8), p>0.99; IL-1β vs. TNF-α, p>0.99; IL-1β vs. IL-6, p=0.14; TNF-α vs. IL-6. p>0.99 |
| Fig. 6f | Kruskal-Wallis test with uncorrected Dunn's multiple comparisons test |
|  | N=3 for each groups; Naïve (n=9) vs. IL-1β (n=10), p>0.99; Naïve vs. TNF-α (n=7), p>0.99; Naïve vs. IL-6 (n=8), p>0.99; IL-1β vs. TNF-α, p>0.99; IL-1β vs. IL-6, p=0.42; TNF-α vs. IL-6. p>0.99 |
| Fig. 6i | unpaired t-test, two-tailed |
|  | CTR (n=6) vs. IL-1β (n=4), p=0.005, t=3.838, df=8 |
|  |  |
| Fig. 6j | two-way ANOVA with Tukey's multiple comparisons test |
|  | CTR (n=6), Familiar vs. Novel, p=0.04; IL-1β (n=4), Familiar vs. Novel, p=0.31, effect of group F(1, 8)=9.213, p=0.02 |
| Fig. 6l | unpaired t-test, two-tailed |
|  | CTR (N=3, n=78) vs. IL-1β (N=3, n=82), p<0.001, t=12.59, df=158 |
| Fig. 6m | unpaired t-test, two-tailed |
|  | CTR (N=3, n=78) vs. IL-1β (N=3, n=82), p<0.001, t=5.105, df=158 |
| Fig. 6n | unpaired t-test, two-tailed |
|  | CTR (N=3, n=78) vs. IL-1β (N=3, n=82), p<0.001, t=5.825, df=158 |
| Fig. 6o | unpaired t-test, two-tailed |
|  | CTR (N=3, n=78) vs. IL-1β (N=3, n=82), p<0.001, t=5.825, df=158 |
| Fig. 7c | one-way ANOVA with Tukey's multiple comparisons test |
|  | CTR (N=5, n=21) vs. CIA (N=5, n=21), p<0.001; CTR vs. CIA+IL-1ra (N=3, n=6), p=0.79; CIA vs. CIA+IL-1ra, p=0.004; F (2,45)=11.10, p<0.001 |
| Fig. 7d | one-way ANOVA with Tukey's multiple comparisons test |
|  | CTR (N=3, n=8) vs. CIA (N=3, n=7), p=0.01; CTR vs. CIA+IL-1ra (N=3, n=6), p=0.73; CIA vs. CIA+IL-1ra, p=0.01; F (2,17)=7.20, p=0.005 |
| Fig. 7e | one-way ANOVA with Tukey's multiple comparisons test |
|  | CTR (N=3, n=8) vs. CIA (N=3, n=7), p=0.03; CTR vs. CIA+IL-1ra (N=3, n=6), p=0.78; CIA vs. CIA+IL-1ra, p=0.16; F (2,18)=4.001, p=0.04 |
| Fig. 7f | one-way ANOVA with Tukey's multiple comparisons test |
|  | CTR (N=5, n=21) vs. CIA (N=5, n=21), p<0.001; CTR vs. CIA+IL-1ra (N=3, n=6), p<0.001; CIA vs. CIA+IL-1ra, p=0.24; F (2,45)=16.10, p<0.001 |
| Fig. 7g | one-way ANOVA with Tukey's multiple comparisons test |
|  | CTR (N=5, n=21) vs. CIA (N=5, n=21), p=0.74; CTR vs. CIA+IL-1ra (N=3, n=6), p=0.94; CIA vs. CIA+IL-1ra, p=0.69; F (2,45)=0.4654, p=0.63 |
| Supplementary Fig. 1 | one-way ANOVA with Tukey's multiple comparisons test |
|  | CTR (n=19) vs. CIA (n=19), p<0.001; CTR vs. CIA+KDS2010 10 mpk (n=15), p=0.009; CIA vs. CIA+KDS2010 10mpk, p=0.008; F (2,42)=19.88, p<0.001 |
| Supplementary Fig. 2b | one-way ANOVA with Tukey's multiple comparisons test |
|  | CTR (n=4) vs. CIA (n=3), p=0.03; CTR vs. CIA+KDS2010 (n=3), p=0.57; CIA vs. CIA+S.KDS2010, p=0.01; F (2,7)=8.782, p=0.01 |
| Supplementary Fig. 2c | two-way ANOVA with Tukey's multiple comparisons test |
|  | CTR (n=4), Familiar vs. Novel, p=0.02; CIA (n=3), Familiar vs. Novel, p=0.84; CIA+S.KDS2010 10mpk (n=3), p=0.006, effect of group F(1, 45)=14.40, p<0.001 |
| Supplementary Fig. 3b | one-way ANOVA with Tukey's multiple comparisons test |
|  | CTR (n=8) vs. CIA (n=8), p=0.92; CTR vs. CIA+KDS2010 (n=8), p=0.92; CIA vs. CIA+KDS2010, p>0.99; F (2,21)=0.1026, p=0.90 |
| Supplementary Fig. 4b | one-way ANOVA with Tukey's multiple comparisons test |
|  | CTR (n=4) vs. CIA (n=4), p=0.64; CTR vs. CIA+KDS2010 (n=4), p>0.99; CIA vs. CIA+S.KDS2010, p=0.64; F (2,9)=0.5725, p=0.58 |
| Supplementary Fig. 5b | one-way ANOVA with Tukey's multiple comparisons test |
|  | CTR (n=69) vs. CIA (n=60), p<0.001; CTR vs. CIA+KDS2010 (n=44), p=0.12; CIA vs. CIA+KDS2010, p<0.001; F (2,170)=15.60, p<0.001 |
| Supplementary Fig. 5c | one-way ANOVA with Tukey's multiple comparisons test |
|  | CTR (n=69) vs. CIA (n=60), p=0.60; CTR vs. CIA+KDS2010 (n=44), p=0.26; CIA vs. CIA+KDS2010, p=0.78; F (2,170)=1.281, p=0.28 |
| Supplementary Fig. 5d | one-way ANOVA with Tukey's multiple comparisons test |
|  | CTR (n=69) vs. CIA (n=60), p=0.46; CTR vs. CIA+KDS2010 (n=44), p=0.62; CIA vs. CIA+KDS2010, p=0.13; F (2,170)=1.960, p=0.14 |
| Supplementary Fig. 5e | one-way ANOVA with Tukey's multiple comparisons test |
|  | CTR (n=6) vs. IL-1β (20ng/ml) (n=6), p=0.04; CTR vs IL-1β (20ng/ml)+KDS2010 (n=3), p>0.99; CTR vs. IL-1β (60ng/ml) (n=6), p=0.01; CTR vs. IL-1β (60ng/ml)+KDS2010 (n=3), p=0.92; IL-1β (20ng/ml) vs. IL-1β (20ng/ml)+KDS2010, p=0.08; IL-1β (20ng/ml) vs. IL-1β (60ng/ml), p=0.99; IL-1β (20ng/ml) vs IL-1β (60ng/ml)+KDS2010, p=0.43; IL-1β (20ng/ml)+KDS2010 vs. IL-1β (60ng/ml), p=0.04; IL-1β (20ng/ml)+KDS2010 vs. IL-1β (60ng/ml)+KDS2010, p=0.90; IL-1β (60ng/ml) vs. IL-1β (60ng/ml)+KDS2010 (p=0.24), F (4,19)=5.459, p=0.004 |
